# Prenatal n-3 long-chain fatty acid status and offspring metabolic health in early and mid-childhood: results from Project Viva

**DOI:** 10.1038/s41387-018-0040-2

**Published:** 2018-05-25

**Authors:** Ekaterina Maslova, Sheryl L. Rifas-Shiman, Sjurdur F. Olsen, Matthew W. Gillman, Emily Oken

**Affiliations:** 1 Centre for Fetal Programming, Department of Epidemiology Research, Statens Serum Institut, Copenhagen, Denmark; 2 Department of Primary Care and Public Health, Imperial College London, London, UK; 3 Danish Diabetes Academy, Odense, Denmark; 4 Division of Chronic Disease Research Across the Lifecourse, Department of Population Medicine, Harvard Medical School and Harvard Pilgrim Health Care Institute, Boston, MA USA; 5 Department of Nutrition, Harvard T.H. Chan School of Public Health, Boston, MA USA

## Abstract

Higher maternal and biomarker levels of n-3 long-chain polyunsaturated fatty acids (LCPUFAs) have been associated with improved perinatal outcomes and may also influence offspring metabolic health. Past studies were not powered to examine metabolic outcomes and few have specifically targeted metabolically vulnerable populations. We examined the associations of prenatal n-3 LCPUFA status with markers of metabolic health in early and mid-childhood in the full population as well as stratified by maternal glucose tolerance. Our data consisted of 1418 mother–child dyads from Project Viva, a longitudinal, prospective pre-birth cohort enrolled in eastern Massachusetts. We assessed maternal dietary intake of fish and n-3 LCPUFA in mid-pregnancy using a validated food frequency questionnaire. N-3 LCPUFA levels were quantified in maternal second trimester and umbilical cord plasma using liquid-gas chromatography. We assessed offspring anthropometry, adiposity, and blood pressure at early (median age: 3.2 years) and mid-childhood (median age: 7.7 years); and assayed blood samples collected at these visits for metabolic biomarkers. We report here multivariable effect estimates and 95% CI. Early childhood BMI *z*-score was on average 0.46 (1.03) units and waist circumference 51.3 (3.7) cm. At mid-childhood these measures were 0.39 (1.00) units and 60.0 (8.3) cm, respectively. Higher cord plasma DHA levels were associated with lower BMI *z*-score ((Q)uartile 4 vs. Q1: −0.21, 95% CI: −0.38, −0.03), waist circumference (Q4 vs. Q1: −0.63, 95% CI: −1.27, 0.00 cm), and leptin levels (Q4 vs. Q1: −0.36, 95% CI: −0.77, 0.05 ng/mL) in early childhood. These associations were strongest and reached significance in offspring of women with isolated hyperglycemia vs. better or worse glycemic status. Higher maternal DHA + EPA (Q4 vs. Q1: −1.59, 95% CI: −2.80, −0.38 μg/mL) and fish (≥3 vs. 0 portions/week: −2.18, 95% CI: −3.90, −0.47 μg/mL) intake was related to lower adiponectin in early childhood. None of these associations persisted with mid-childhood outcomes. We did not find associations with any of the other outcomes. This study supports early and possibly transient effects of prenatal n-3 LCPUFA status on anthropometric measures and adipokine levels. It also raises the possibility that offspring of women with isolated hyperglycemia derive the most benefits from higher n-3 LCPUFA status.

## Introduction

N-3 long-chain polyunsaturated fatty acids (LCPUFAs) eicosapentaenoic acid (EPA) and docosahexaenoic acid (DHA) are essential polyunsaturated fatty acids found primarily in fish and seafood. They can also be synthesized from alpha-linolenic acid found in vegetable oils and nuts, but at a relatively low efficiency. N-3 LCPUFAs are recommended to pregnant women to sustain the rapid brain development in the fetus. In non-pregnant adults, these fatty acids have been associated with better cardiometabolic profiles in both randomized trials and large, longitudinal cohorts in European, US, and Asian populations^1^. Some evidence now suggests that prenatal exposure to these fatty acids may also influence developmental pathways toward better offspring metabolic health. For example, animal studies have shown that offspring to dams fed diets supplemented with fish oil or n-3 LCPUFA had lower levels of adiposity^2–5^, insulin resistance^3–6^, and cholesterol and triglyceride levels^4^ when compared to dams fed diets low in n-3 but rich in saturated fats or n-6 fatty acids.

Human data have been largely focused on anthropometry and body composition measures, while other metabolic outcomes being less explored. Some of these studies have found associations of higher maternal n-3 status with lower cord blood insulin levels^7^, higher lean mass^8^, and lower adiposity and^9^ body mass index (BMI) *z*-scores^10^ in early to mid-childhood; while others showed no associations^11–18^. Reviews and meta-analyses of randomized clinical trials have concluded that there is little evidence to support an effect of pre- or perinatal n-3 fatty acid supplementation on offspring body composition measures^19–21^. However, many of these trials were limited by post hoc analysis of childhood adiposity as they were originally designed to evaluate pregnancy outcomes and infant neurodevelopment, inadequate blinding, high attrition rates, diverse timing of outcome assessment, and not correcting for age and sex. One subsequent trial that was specifically designed to address some of these limitations found no effect of n-3 LCPUFA supplementation from ≤ 15 weeks of gestation through lactation on offspring body composition at 12 months^22^. In a recent pooled analysis of 15 European and US cohorts, the authors found, contrary to hypothesis, that maternal fish intake ≥3 times/week vs. ≤ 1 times/week was associated with higher odds of offspring overweight/obesity at age 4^23^, but that study did not include estimates of fatty acid intake, other cardiometabolic outcomes, or direct measures of adiposity.

With rising obesity levels, also among populations of reproductive age, more women are entering pregnancy in suboptimal metabolic health. These women are at higher risk of pregnancy complications, including impaired glucose tolerance and gestational diabetes mellitus (GDM)^24^. Furthermore, offspring of mothers with abnormal gestational glycemia are at greater risk of obesity and diabetes themselves^24–26^. These high-risk children may be in particular need of optimal prenatal nutrition, including n-3 LCPUFA. N-3 LCPUFAs have been found to have insulin-sensitizing effects^27^ that could potentially reduce offspring exposure to hyperglycemia, which could be especially relevant considering altered LCPUFA metabolism in women affected by GDM^28–30^.

In this study, we aimed at examining the associations of maternal n-3 LCPUFA status and intake in pregnancy with offspring metabolic outcomes in early and mid-childhood using data from the prospective, longitudinal pre-birth cohort Project Viva. We have examined this association in a prior study with early childhood data where DHA + EPA exposure was associated with lower sum of skinfold and risk of obesity at age 3 years^31^. We now study this question with additional cord plasma data and additional outcomes in mid-childhood that include direct assessment of offspring adiposity in the full cohort and stratified by maternal glucose tolerance status^25,26^. We hypothesized that higher n-3 LCPUFA status and intake will be associated with an improved offspring metabolic profile, and that the effect sizes will be larger in women with impaired glucose tolerance and GDM.

## Methods

### Study design and population

Between 1999 and 2002 we recruited pregnant women at eight obstetric offices of Atrius Harvard Vanguard Medical Associates, a multispecialty group practice in eastern Massachusetts. Recruitment and retention strategies have been described elsewhere^32^. In brief, women were recruited during their first prenatal visit (median: 9.9 weeks’ gestation). Eligibility criteria included being able to answer questions in English, planning to stay in the area until after delivery, presenting for prenatal care before 22 weeks’ gestation, and carrying a singleton pregnancy. Follow-up visits took place in the second trimester, at the birth admission, in infancy (median age: 6.3 months), early childhood (median age: 3.2 years), and mid-childhood (median age: 7.7 years). We mailed questionnaires to participants in the years between the visits.

**Fig. 1:**
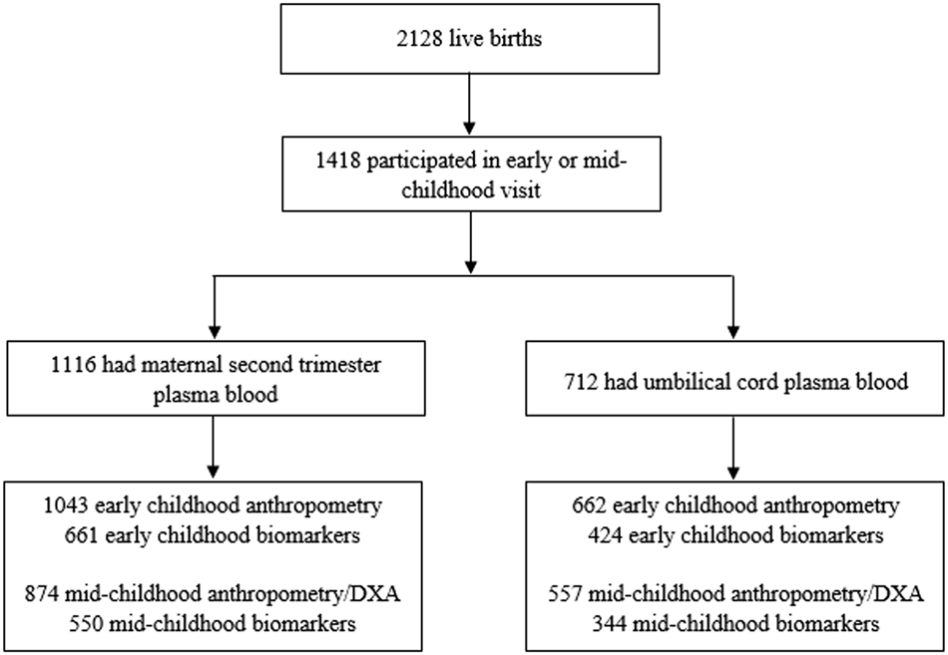
Flow chart of mother–child pairs in the Project Viva .

In this analysis, we included mother–offspring dyads with available exposure and outcome data. A total of 2128 women delivered a live infant, and 1418 completed either an early or a mid-childhood visit (Fig. 1). Of the 1418, 1116 women had blood samples from the second trimester visit and 712 had cord blood collected at delivery. The number of participants available for analysis varied according to the specific exposure and outcome with the lowest number of participants available for the cord blood biomarker-mid-childhood metabolic biomarker analyses (*n* = 344).

Mothers included (*n* = 1418) vs. excluded (*n* = 710) in this analysis were more likely to be white (68.8% vs. 61.6%), college graduates (68.2% vs. 57.5%), have an annual household income of above $70 000 (60.3% vs. 53.0%), and never to have smoked cigarettes (69.3% vs. 66.8%), and less likely to have GDM (4.8% vs. 7.6%). They breastfed for a longer duration (6.2 vs. 4.5 months). We also observed small differences for maternal and cord plasma n-3 LCPUFA, second trimester fish intake, maternal age, parity, marital status, pre-pregnancy BMI, gestational weight gain, and offspring birth weight and gestational age (data not shown).

The institutional review boards of participating institutions approved the study, and all procedures were in accordance with ethical standards for human experimentation. All women provided written informed consent for themselves and on behalf of their children. Starting with the mid-childhood visit, children also provided verbal assent.

### Exposure assessment

We assessed prenatal n-3 LCPUFA status through maternal diet, maternal blood levels, and cord blood level.

#### Dietary intake

Mothers reported dietary intake via validated semi-quantitative food frequency questionnaires (FFQs)^33,34^. Diet in the past 3 months was assessed at the second trimester visit (gestational weeks 26–28). In this cohort, we previously reported strong correlations between diet and fatty acid biomarkers^34^. The FFQ included four questions on intake of canned tuna fish (3–4 ounces); shrimp, lobster, scallops, and clams (1 serving); dark-meat fish (e.g., mackerel, salmon, sardines, bluefish, and swordfish (3–5 ounces)); and other fish (e.g., cod, haddock, and halibut (3–5 ounces)). Response options ranged from “never/<1 per month” to “1 or more servings per day” across six categories. To calculate total fish intake, we totaled the responses from the four questions.

We quantified intake of two n-3 LCPUFAs, EPA and DHA, by multiplying the frequency of consumption of each food item by the nutrient content for the specified portion size and summing the nutrient intake for all food items. Information on nutrient estimates was retrieved from the Harvard nutrient-composition database, which is based on the US Department of Agriculture publications and continually supplemented by other published sources and personal communications from laboratories and manufacturers^35^. We energy-adjusted estimates for these nutrients using the nutrient residuals method^36^. We summed up dietary EPA (mean (SD): 0.05 (0.07) g/day) and DHA (mean (SD): 0.10 (0.09) g/day) given the low levels of EPA intake and because we could not examine differential bioactivity with intake as it was not possible to determine the amount of EPA metabolized into DHA.

#### Maternal and umbilical blood

At the second trimester visit, we collected blood samples into vacutainer tubes containing ethylenediaminetetraacetic acid (EDTA). The EDTA tubes were centrifuged at 2000 rpm for 10 min at 4°C to separate plasma from erythrocytes, which was washed with chilled saline. We stored erythrocyte and plasma aliquots at −70°C, but did not keep any whole blood. We collected umbilical cord blood by venipuncture after delivery of the infant, and stored plasma at −70°C. We did not retain cord erythrocytes.

Maternal and cord plasma fatty acids levels (in μg/ml) were measured using liquid-gas chromatography. Analytic methods have good within-run precision (coefficient of variance < 5.4%) and been previously validated^37,38^. For this analysis, we used DHA and EPA, the two n-3 LCPUFAs that are found in highest concentrations in fish. We examined them separately for potentially differential bioactivity.

### Outcome assessment

We included anthropometric, adiposity, and metabolic outcomes from the early and mid-childhood visits.

#### Early childhood (median age: 3.2 years)

From this visit we considered the following outcomes: anthropometric BMI; waist circumferences); adiposity (skinfold measurements); and adipokines (leptin and adiponectin). We measured height and weight of offspring using a calibrated stadiometer (Shorr Productions, Olney, MD) and scale (Seca model 881, Seca Corp, Hanover, MD). We calculated BMI as kg/m^2^ and determined age- and sex-specific BMI *z*-scores using the US national reference data^39^. Research assistants measured waist circumference using a Hoechstmass non-stretchable measuring tape (Hoechstmass Balzer GmbH, Sulzbach, Germany). We quantified subscapular (SS) and triceps (TR) skinfold thicknesses using Holtain calipers (Holtain, Cross-well, UK), and calculated the sum (SS + TR) and the ratio ([SS:TR] × 100) of skinfolds. BMI and SS + TR represent overall adiposity and SS:TR central or truncal adiposity^40^. Research assistants used standardized techniques^41^ and took part in biannual in-service training to ensure measurement validity^42^. Inter- and intra-rater measurement errors were within published reference ranges for all included measurements^43^. Blood pressure (BP) was measured up to five times for each offspring, at 1-min intervals, using biannually calibrated Dinamap Pro 100 or Pro 200 (Critikon Inc.) automated BP monitors. We included systolic BP in this analysis as it is a better predictor of future cardiovascular risk factors^44^. We measured plasma leptin (ng/mL) and adiponectin (µg/mL) concentrations with a radioimmunoassay (Linco Research, St Charles, MO).

The early outcomes were part of a prior paper^31^, but we re-examined them here with additional cord plasma data (*n* = 712 vs. 302) assayed in a different laboratory and additionally stratified on maternal glycemic status (please see next section for definition of glycemic status).

#### Mid-childhood (median age: 7.7 years)

In addition to the same outcomes we measured at early childhood, in mid-childhood we also included the following: body composition (fat and fat-free mass) and metabolic (fasting insulin (µU/mL); fasting glucose (mg/dL); homeostatic model assessment for insulin resistance (HOMA-IR); total cholesterol (mg/dL); high-density lipoprotein cholesterol (HDL-C; mg/dL); triglycerides (mg/dL)) measures; and a metabolic risk score. Body weight was measured to nearest 0.1 kg using a different electronic scale (Tanita, Arlington Heights, IL). We measured body composition using dual-energy radiograph absorptiometry (DXA) (Hologic model Discovery A, Hologic, Bedford, MA, USA) and employed Hologic software version 12.6 for scan analysis. From these data, we derived percent body fat, trunk fat mass index (TFMI, kg/m^2^), fat mass index (FMI, kg/m^2^), fat-free mass index (FFMI,  kg/m^2^), and peripheral fat mass index  (PFMI,  kg/m^2^). Plasma fasting insulin was quantified using an electrochemiluminescence immunoassay on the Roche E Modular system. We assessed fasting glucose from a lithium heparin tube enzymatically using Roche Diagnostics reagents (Roche Diagnostics, Indianapolis, IN) and calculated HOMA-IR using ([(fasting plasma insulin (µU/mL) × fasting plasma glucose(mg/dL))/405])^45^. We measured lipids (total cholesterol, triglycerides, and HDL-C) enzymatically with correction for endogenous glycerol. We derived a mid-childhood metabolic risk score as the mean of five sex-specific internal *z*-scores for systolic BP, waist circumference, log-transformed HOMA-IR, triglycerides, and HDL-C (scaled inversely)^46–48^.

### Covariates and effect modifiers

We considered possible biological and social causal pathways and decided a priori to include the following covariates: household income (> and ≤ $70 000/year); maternal age (continuous); parity (nulliparous and multiparous); maternal education (≥ and <college graduate); pre-pregnancy BMI (continuous); smoking in pregnancy (never, former, and smoker); and offspring age and sex. Race/ethnicity was not included as we have previously shown that maternal fatty acid status was similar across race/ethnicity strata;^34^ in a sensitivity analysis, adding race/ethnicity to the models did not change the results. Intermediary covariates (gestational weight gain, birth weight, and gestational age) were excluded to avoid overadjustment.

Women in the study population were routinely screened for abnormal glycemia with a non-fasting 50 g oral glucose challenge test at 24–28 weeks’ gestation^26^. One-hour post-load glucose levels ≥140 mg/dL resulted in referral for a fasting 3 h, 100 g oral glucose tolerance test (OGTT). Normal levels was classified as blood glucose < 95 mg/dL at baseline, <180 mg/dL at 1 h, <155 mg/dL at 2 h, and <140 mg/dL at 3 h (Carpenter and Coustan criteria^49^). We created four categories of gestational glycemia. Women with normal glucose tolerance had levels < 140 mg/dL on the glucose challenge and thus were not refereed to an OGTT. We defined isolated hyperglycemia as a challenge test ≥140 mg/dL, but normal OGTT results; gestational impaired glucose tolerance (GIGT) as a failed challenge test and 1 abnormal OGTT results; and GDM if ≥2 abnormal OGTT results^26^. We include four groups to examine glucose intolerance on a spectrum. Women reported their dietary intake and provided blood samples at the time of the GDM screening and before a diagnosis was confirmed.

### Statistical analysis

We conducted the analyses for the full cohort and separately within the four gestational glycemia strata. Quantification of *P*-values for interaction did not reveal any interactions <0.05. In descriptive analyses, we examined the mean (SD) for the continuous variables and percentages for the categorical variables. We used Pearson correlation coefficients to quantify correlations between biomarkers. We examined multivariable associations of prenatal exposures with offspring anthropometry and metabolic variables using linear regression models. We included the prenatal exposures both as continuous and categorical variables to account for potential nonlinear associations. For the exposures modeled as continuous variables, we set the unit of exposure as internal *z*-score for the fatty acid biomarkers, 100 mg/day for the fatty acid intake, and portions/week for fish intake. We categorized the fatty acid biomarkers and intake using quartiles. Fish intake was assessed as 0, >0 to <3, and ≥3 portions/week, in line with current recommendations by the U.S. Food and Drug Administration^50^. We adjusted the models in two stages, first for offspring age and sex, and second for all a priori considered parental and offspring covariates. As the exposures and outcome measures we studied were closely related, we examined the results for strength of association and consistency rather than employing more conservative adjustments for multiple comparisons.

A common issue in large longitudinal studies is missing data on one or more covariate. We used PROC MI (SAS version 9.4, Cary, NC) and imputed 50 values for each missing observation to create 50 “completed” datasets, including all 2128 mother–offspring pairs. Following imputation, we combined the multivariable modeling estimates using PROC MI ANALYZE. In adherence with Project Viva protocols, we set our analytic sample sizes based on those who were eligible to have exposures and outcomes. Characteristics were similar in the unimputed and imputed datasets (data not shown).

We performed all analyses using Statistical Analyses System software (release 9.4; SAS Institute, Cary, NC). Statistical significance was defined at *P* < 0.05.

### Code availability

No code availability at this time.

## Results

### Study population

Table 1 shows the characteristics of the study population. Mean (SD) maternal age was 32.1 (5.2) years and a majority of women were multiparous (52.7%), white (68.8%), college-educated (68.2%), and had an annual household income above $70 000 (60.3%). Mean (SD) pre-pregnancy BMI was 24.8 (5.3) kg/m^2^ and gestational weight gain was 15.6 (5.4) kg. Most women reported never smoking during pregnancy (89.1%). The proportion of women classified as having GDM was 4.8%, while 3.2% had GIGT and 8.6% isolated hyperglycemia.

**Table 1:** Parental and child characteristics in Project Viva (*n* = 1418)

**Characteristics**	*N* (%) or mean (SD)
*Mother*	
Age at enrollment, years	32.1 (5.2)
Nulliparous, %	
Yes	671 (47.3)
Race/ethnicity	
Black	210 (14.8)
Hispanic	96 (6.8)
White	975 (68.8)
Other	137 (9.7)
≥College graduate, %	
Yes	967 (68.2)
Married or cohabitating	
Yes	1300 (91.7)
Household income > $70 000/year	
Yes	854 (60.3)
Pre-pregnancy BMI, kg/m^2^	24.8 (5.3)
Pregnancy weight gain, kg	15.6 (5.4)
Pregnancy smoking status	
Never	983 (69.3)
Former	280 (19.8)
Smoked during pregnancy	155 (10.9)
Glucose tolerance status	
GDM	68 (4.8)
GIGT	45 (3.2)
IH	122 (8.6)
Normal	1183 (83.4)
*Child*	
Sex, %	
Male	728 (51.3)
Race/ethnicity, %	
Black	214 (15.1)
Hispanic	69 (4.9)
White	924 (65.2)
Other	210 (14.8)
Birth weight, gm	3481 (570)
Gestation length, weeks	39.5 (1.8)
Breastfeeding duration, months	6.2 (4.6)
*Early childhood*	
Age, years	3.3 (0.4)
BMI, kg/m^2^	16.5 (1.5)
BMI *z*-score	0.46 (1.03)
Waist circumference, cm	51.3 (3.7)
Sum of skinfolds (SS + TR), mm	16.7 (4.3)
Ratio of skinfolds (SS:TR × 100)	64.5 (16.0)
SBP, mm Hg	92.2 (10.7)
Adiponectin, μg/mL	22.3 (5.6)
Leptin, ng/mL	1.9 (1.9)
*Mid-childhood*	
Age, years	8.0 (0.9)
BMI, kg/m^2^	17.2 (3.0)
BMI *z*-score	0.39 (1.00)
Waist circumference, cm	60.0 (8.3)
Sum of skinfolds (SS + TR), mm	19.9 (9.8)
Ratio of skinfolds (SS:TR × 100)	70.5 (18.9)
SBP, mm Hg	94.6 (8.7)
DXA percent fat	24.6 (6.3)
DXA FMI, kg/m^2^	4.4 (1.9)
DXA FFMI, kg/m^2^	13.0 (1.5)
DXA trunk FMI, kg/m^2^	1.5 (0.9)
DXA peripheral FMI, kg/m^2^	2.5 (1.1)
Fasting insulin, μU/ml	7.8 (6.4)
Fasting glucose, mg/dL	94.4 (15.0)
HOMA-IR	1.8 (1.7)
Total cholesterol, mg/dL	160.4 (27.7)
Triglyceride, mg/dL	57.9 (25.6)
HDL-C, mg/dL	57.2 (13.6)
Adiponectin, μg/mL	15.5 (8.8)
Leptin, ng/mL	6.0 (7.3)
Metabolic risk *z*-score	0.00 (0.62)

*BMI* body mass index, *DXA* dual-energy radiograph absorptiometry, *GDM* gestational diabetes mellitus, *GIGT* gestational impaired glucose intolerance, *HDL-C* high-density lipoprotein cholesterol, *HOMA-IR* homeostatic model assessment for insulin resistance, *IH* isolated hyperglycemia, *SBP* systolic blood pressure, *SS* subscapular, *TR* triceps

Early childhood BMI *z*-score was on average 0.46 (1.03) and waist circumference 51.3 (3.7) cm (Table 1). At mid-childhood, these measures were 0.39 (1.00) and 60.0 (8.3) cm, respectively. The ratio of SS:TR × 100 was 64.5% (16.0%) in early childhood and 70.5% (18.9%) in mid-childhood. Leptin was higher and adiponectin lower in mid- vs. early childhood. Systolic BP remained relatively stable across the two measuring points.

Mean (SD) of second trimester maternal plasma EPA and DHA levels were 10.9 (8.8) and 88.9 (37.9) μg/mL, respectively. The corresponding levels in cord plasma were 1.7 (1.6) and 33.6 (20.2) μg/mL. The average fish intake was 1.6 (1.5) portions/week. The correlations (Pearson’s *r*) between maternal and cord plasma EPA and DHA were 0.26 and 0.11, respectively.

### Prenatal n-3 LCPUFA status, adiposity, and metabolic health in early childhood

We found the most consistent inverse associations for EPA and DHA levels with BMI *z*-scores, with the stronger results present for cord plasma DHA (Table 2). Compared to offspring in the lowest quartile of cord plasma DHA levels, offspring in highest quartile had 0.21 units lower (95% confidence interval (CI): −0.38, −0.03) BMI *z*-score. This association was stronger in offspring born to women with isolated hyperglycemia (Q4 vs. Q1: −0.65, 95% CI: −1.21, −0.10) and showed a dose response relation in both the full cohort and the isolated hyperglycemia subgroup. Cord plasma DHA was also weakly related to a lower offspring waist circumference (Q4 vs. Q1: −0.63, 95% CI: −1.27, 0.00 cm) (Table 3). Furthermore, we found that both cord plasma EPA (Q4 vs. Q1: −0.36, 95% CI: −0.78, 0.06 ng/mL) and DHA (Q4 vs. Q1: −0.36, 95% CI: –0.77, 0.05 ng/mL) were associated with lower offspring leptin levels in the full cohort, but CIs excluded the null only for DHA among offspring to women with isolated hyperglycemia (Q4 vs. Q1: −1.27, 95% CI: −2.53, −0.02 ng/mL) (Supplement Table 1). There was a suggestive inverse association of cord plasma DHA with offspring SS:TR × 100 ratio in the total population, but CIs were wide (Q4 vs. Q1: −2.14%, 95% CI: −4.98, 0.70%) (data not shown).

**Table 2:** Multivariable association of prenatal n-3 LCPUFA status and intake with offspring early childhood BMI *z*-scores, overall and stratified by glucose tolerance status

	**Total**	**Total**	**GDM**	**GIGT**	**IH**	**Normal**
**Exposure**	**Age- and sex-adjusted** ***β*** **(95% CI)**	MV-adjusted^a^ ***β*** **(95% CI)**	**MV-adjusted** ^**a**^ ***β*** **(95% CI)**	MV-adjusted^a^ ***β*** **(95% CI)**	MV-adjusted^a^ ***β*** **(95% CI)**	MV-adjusted^a^ ***β*** **(95% CI)**
*Continuous exposure*						
Second T plasma EPA (per *z*-score)	−0.05 (−0.12, 0.01)	−0.04 (−0.10, 0.03)	−0.21 (−0.65, 0.24)	−0.45 (−1.92, 1.01)	−0.20 (−0.45, 0.06)	−0.01 (−0.08, 0.06)
Second T plasma DHA (per *z*-score)	−0.04 (−0.11, 0.03)	−0.03 (−0.10, 0.05)	−0.44 (−0.87, −0.02)**	−0.03 (−0.78, 0.71)	−0.02 (−0.26, 0.22)	0.00 (−0.08, 0.07)
Cord plasma EPA (per *z*-score)	−0.09 (−0.17, 0.00)**	−0.05 (−0.14, 0.03)	−0.08 (−0.65, 0.50)	0.24 (−0.99, 1.48)	−0.30 (−0.57, −0.04)**	−0.04 (−0.13, 0.06)
Cord plasma DHA (per *z*-score)	−0.12 (−0.20, −0.03)**	−0.09 (−0.18, −0.01)**	−0.07 (−0.62, 0.47)	0.29 (−0.64, 1.23)	−0.30 (−0.52, −0.07)**	−0.08 (−0.18, 0.01)
DHA + EPA intake (100 mg/day)	−0.02 (−0.06, 0.02)	−0.01 (−0.05, 0.03)	0.04 (−0.15, 0.23)	−0.39 (−0.77, −0.01)**	−0.05 (−0.20, 0.11)	−0.01 (−0.05, 0.03)
Fish intake (port/week)	0.00 (−0.04, 0.04)	0.01 (−0.03, 0.05)	0.01 (−0.18, 0.20)	−0.39 (−0.94, 0.17)	−0.02 (−0.16, 0.12)	0.01 (−0.03, 0.05)
*Categorical exposure*						
Second T plasma EPA						
Q2 vs. Q1	−0.09 (−0.26, 0.09)	−0.07 (−0.24, 0.10)	−0.06 (−1.17, 1.06)	−0.46 (−2.10, 1.19)	0.01 (−0.64, 0.65)	−0.09 (−0.27, 0.10)
Q3 vs. Q1	−0.16 (−0.34, 0.01)	−0.15 (−0.32, 0.02)	−0.83 (−1.86, 0.20)	−0.20 (−1.25, 0.85)	−0.02 (−0.60, 0.57)	−0.14 (−0.32, 0.05)
Q4 vs. Q1	−0.14 (−0.31, 0.03)	−0.10 (−0.27, 0.07)	−0.98 (−2.13, 0.17)	0.00 (−1.76, 1.77)	−0.26 (−0.84, 0.33)	−0.06 (−0.25, 0.12)
Second T plasma DHA						
Q2 vs. Q1	0.01 (−0.18, 0.19)	0.01 (−0.16, 0.19)	0.36 (−0.69, 1.40)	0.75 (−0.76, 2.26)	−0.06 (−0.65, 0.53)	−0.02 (−0.21, 0.17)
Q3 vs. Q1	−0.03 (−0.20, 0.14)	−0.02 (−0.19, 0.14)	−0.68 (−1.73, 0.37)	−0.03 (−1.25, 1.19)	−0.08 (−0.67, 0.51)	−0.01 (−0.19, 0.18)
Q4 vs. Q1	−0.10 (−0.28, 0.07)	−0.06 (−0.23, 0.11)	−0.75 (−1.73, 0.24)	0.07 (−1.14, 1.28)	−0.03 (−0.69, 0.62)	−0.04 (−0.23, 0.15)
Cord plasma EPA						
Q2 vs. Q1	−0.01 (−0.20, 0.18)	−0.01 (−0.20, 0.17)	0.23 (−1.16, 1.62)	0.37 (−0.96, 1.70)	−0.09 (−0.75, 0.57)	0.01 (−0.19, 0.21)
Q3 vs. Q1	−0.06 (−0.25, 0.13)	−0.04 (−0.23, 0.14)	−0.30 (−1.30, 0.71)	0.51 (−0.93, 1.94)	0.05 (−0.55, 0.66)	−0.06 (−0.27, 0.14)
Q4 vs. Q1	−0.20 (−0.38, −0.02)**	−0.15 (−0.33, 0.02)	0.11 (−1.10, 1.31)	0.16 (−1.64, 1.95)	−0.56 (−1.15, 0.03)	−0.12 (−0.32, 0.07)
Cord plasma DHA						
Q2 vs. Q1	0.02 (−0.18, 0.21)	−0.01 (−0.19, 0.18)	0.03 (−1.09, 1.14)	0.59 (−0.71, 1.89)	0.14 (−0.52, 0.81)	−0.02 (−0.22, 0.18)
Q3 vs. Q1	−0.12 (−0.31, 0.06)	−0.12 (−0.30, 0.06)	−0.56 (−1.63, 0.51)	0.53 (−1.09, 2.16)	−0.01 (−0.67, 0.65)	−0.13 (−0.33, 0.06)
Q4 vs. Q1	−0.23 (−0.41, −0.05)**	−0.21 (−0.38, −0.03)**	0.41 (−0.79, 1.61)	0.23 (−1.27, 1.74)	−0.65 (−1.21, −0.10)**	−0.17 (−0.37, 0.03)
DHA + EPA intake						
Q2 vs. Q1	0.06 (−0.10, 0.23)	0.06 (−0.10, 0.22)	−0.38 (−1.41, 0.64)	−0.66 (−2.04, 0.72)	−0.10 (−0.69, 0.49)	0.10 (−0.07, 0.28)
Q3 vs. Q1	−0.09 (−0.25, 0.08)	−0.08 (−0.25, 0.08)	−0.88 (−1.85, 0.08)	−0.23 (−1.34, 0.88)	−0.41 (−0.94, 0.12)	0.00 (−0.19, 0.18)
Q4 vs. Q1	−0.09 (−0.26, 0.08)	−0.07 (−0.23, 0.09)	−0.45 (−1.62, 0.73)	−1.25 (−2.36, −0.15)**	−0.16 (−0.73, 0.41)	−0.03 (−0.20, 0.15)
Fish intake						
>0 to <3 vs. 0 port/wk)	−0.05 (−0.23, 0.13)	−0.05 (−0.22, 0.13)	−0.28 (−1.17, 0.62)	−0.57 (−1.70, 0.57)	0.01 (−0.64, 0.65)	−0.01 (−0.20, 0.18)
≥3 vs. 0 port/wk)	0.00 (−0.24, 0.23)	0.00 (−0.23, 0.22)	−0.33 (−1.58, 0.91)	−0.14 (−1.69, 1.41)	0.06 (−0.80, 0.92)	0.03 (−0.21, 0.28)

*EPA* eicosapentaenoic acid, *DHA* docosahexaenoic acid, *GDM* gestational diabetes mellitus, *GIGT* gestational impaired glucose intolerance, *IH* isolated hyperglycemia, *port* portion, *Q* quartile, *T* trimester

**P < 0.05

^a^Adjusted for child age and sex + maternal education, age, parity, pre-pregnancy BMI, smoking during pregnancy, and household income

**Table 3:** Multivariable association of prenatal n-3 LCPUFA status and intake with offspring early childhood waist circumference (cm), overall and stratified by glucose tolerance status

	**Total**	**Total**	**GDM**	**GIGT**	**IH**	**Normal**
**Exposure**	**Age- and sex-adjusted** ***β*** **(95% CI)**	MV-adjusted^a^ ***β*** **(95% CI)**	MV-adjusted^a^ ***β*** **(95% CI)**	MV-adjusted^a^ ***β*** **(95% CI)**	MV-adjusted^a^ ***β*** **(95% CI)**	MV-adjusted^a^ ***β*** **(95% CI)**
*Continuous exposure*						
Second T plasma EPA (per *z*-score)	−0.02 (−0.25, 0.21)	0.00 (−0.23, 0.23)	−0.16 (−1.64, 1.31)	−1.37 (−6.25, 3.52)	−0.26 (−1.17, 0.64)	0.06 (−0.18, 0.30)
Second T plasma DHA (per *z*- score)	0.06 (−0.18, 0.31)	0.07 (−0.17, 0.32)	−0.66 (−2.17, 0.84)	−0.43 (−2.98, 2.13)	0.26 (−0.60, 1.12)	0.11 (−0.15, 0.36)
Cord plasma EPA (per *z*-score)	−0.20 (−0.49, 0.10)	−0.12 (−0.42, 0.17)	−0.40 (−2.31, 1.51)	1.86 (−3.08, 6.81)	−0.64 (−1.59, 0.31)	−0.11 (−0.43, 0.21)
Cord plasma DHA (per *z*-score)	−0.31 (−0.61, −0.01)**	−0.26 (−0.55, 0.04)	−0.08 (−1.87, 1.71)	1.54 (−2.17, 5.24)	−0.63 (−1.45, 0.19)	−0.26 (−0.61, 0.08)
DHA + EPA intake (100 mg/day)	−0.04 (−0.17, 0.09)	−0.03 (−0.16, 0.10)	0.22 (−0.47, 0.92)	−0.96 (−2.41, 0.49)	−0.08 (−0.68, 0.52)	−0.04 (−0.18, 0.10)
Fish intake (port/week)	−0.03 (−0.18, 0.11)	−0.04 (−0.18, 0.11)	0.29 (−0.38, 0.97)	−1.10 (−3.08, 0.88)	−0.07 (−0.59, 0.45)	−0.05 (−0.21, 0.11)
*Categorical exposure*						
Second T plasma EPA						
Q2 vs Q1	0.04 (−0.57, 0.65)	0.06 (−0.54, 0.65)	0.26 (−4.16, 4.68)	0.15 (−5.20, 5.50)	1.07 (−1.38, 3.52)	−0.19 (−0.83, 0.45)
Q3 vs. Q1	−0.14 (−0.73, 0.46)	−0.12 (−0.71, 0.46)	−1.88 (−5.77, 2.00)	0.82 (−3.02, 4.65)	0.55 (−1.60, 2.71)	−0.20 (−0.83, 0.44)
Q4 vs. Q1	−0.03 (−0.64, 0.57)	0.04 (−0.57, 0.65)	−1.26 (−5.55, 3.02)	0.74 (−5.64, 7.12)	0.14 (−2.03, 2.32)	0.00 (−0.64, 0.63)
Second T plasma DHA						
Q2 vs. Q1	0.12 (−0.51, 0.74)	0.11 (−0.50, 0.72)	1.92 (−2.08, 5.91)	4.80 (−0.64, 10.24)	0.58 (−1.58, 2.74)	−0.15 (−0.82, 0.51)
Q3 vs. Q1	0.06 (−0.54, 0.65)	0.04 (−0.55, 0.63)	−1.34 (−5.21, 2.53)	−0.12 (−4.12, 3.88)	0.38 (−1.81, 2.57)	0.03 (−0.62, 0.68)
Q4 vs. Q1	0.10 (−0.51, 0.71)	0.18 (−0.43, 0.79)	−0.86 (−4.52, 2.81)	1.33 (−2.77, 5.42)	1.42 (−0.97, 3.81)	0.00 (−0.65, 0.65)
Cord plasma EPA						
Q2 vs. Q1	−0.21 (−0.89, 0.46)	−0.22 (−0.89, 0.45)	1.32 (−3.62, 6.27)	2.28 (−2.53, 7.09)	−1.75 (−4.22, 0.73)	−0.08 (−0.79, 0.63)
Q3 vs. Q1	−0.20 (−0.85, 0.46)	−0.11 (−0.76, 0.54)	−1.21 (−4.86, 2.43)	1.92 (−3.20, 7.04)	0.72 (−1.56, 3.00)	−0.14 (−0.86, 0.58)
Q4 vs. Q1	−0.58 (−1.21, 0.06)	−0.48 (−1.11, 0.15)	1.18 (−3.20, 5.55)	3.43 (−3.39, 10.26)	−1.67 (−3.84, 0.51)	−0.44 (−1.12, 0.23)
Cord plasma DHA						
Q2 vs. Q1	−0.10 (−0.77, 0.58)	−0.16 (−0.82, 0.49)	1.47 (−2.55, 5.48)	2.90 (−1.86, 7.66)	−0.46 (−2.97, 2.04)	−0.20 (−0.89, 0.49)
Q3 vs. Q1	−0.60 (−1.24, 0.04)	−0.57 (−1.20, 0.06)	−2.25 (−5.99, 1.49)	1.76 (−4.06, 7.57)	0.44 (−2.02, 2.91)	−0.58 (−1.25, 0.10)
Q4 vs. Q1	−0.69 (−1.33, −0.05)**	−0.63 (−1.27, 0.00)**	2.32 (−2.06, 6.69)	3.14 (−2.56, 8.84)	−1.95 (−4.00, 0.11)	−0.59 (−1.28, 0.11)
DHA + EPA intake						
Q2 vs. Q1	−0.08 (−0.68, 0.51)	−0.14 (−0.72, 0.44)	−2.82 (−6.51, 0.87)	−4.99 (−9.69, −0.29)**	0.29 (−1.94, 2.51)	0.00 (−0.63, 0.62)
Q3 vs. Q1	−0.53 (−1.12, 0.07)	−0.57 (−1.15, 0.02)	−3.53 (−7.02, −0.03)**	−0.01 (−3.99, 3.98)	−0.81 (−2.82, 1.19)	−0.40 (−1.05, 0.24)
Q4 vs. Q1	−0.40 (−0.99, 0.19)	−0.40 (−0.98, 0.19)	−1.75 (−5.93, 2.43)	−2.87 (−6.90, 1.15)	−0.08 (−2.25, 2.09)	−0.31 (−0.94, 0.31)
Fish intake						
>0 to <3 vs. 0 port/wk	−0.14 (−0.78, 0.50)	−0.15 (−0.78, 0.48)	−1.92 (−5.18, 1.33)	0.06 (−3.91, 4.03)	0.88 (−1.58, 3.33)	−0.16 (−0.84, 0.53)
≥3 vs. 0 port/wk	−0.18 (−1.02, 0.67)	−0.21 (−1.04, 0.62)	−0.62 (−5.15, 3.92)	−0.13 (−5.10, 4.84)	0.41 (−2.80, 3.62)	−0.24 (−1.13, 0.66)

*EPA* eicosapentaenoic acid, *DHA* docosahexaenoic acid, *GDM* gestational diabetes mellitus, *GIGT* gestational impaired glucose intolerance, *IH* isolated hyperglycemia, *port* portion, *Q* quartile, *T* trimester

**P < 0.05

^a^Adjusted for child age and sex + maternal education, age, parity, pre-pregnancy BMI, smoking during pregnancy, and household income

Maternal intake of DHA + EPA (Q4 vs. Q1: −1.59, 95% CI: −2.80, −0.38 μg/mL) and fish (≥3 vs. 0 portions/week: −2.18, 95% CI: −3.90, −0.47 μg/mL) were related to lower offspring adiponectin, showing a dose response (Supplement Table 2). We found no consistent associations of any of the exposures with offspring skinfold measures or systolic BP (data not shown).

### Prenatal n-3 LCPUFA status, adiposity, and metabolic health in mid-childhood

We found that the associations of cord plasma DHA with mid-childhood offspring BMI *z*-score in both the total cohort (Q4 vs. Q1: −0.14 *z*-scores, 95% CI: −0.32, 0.04) and among offspring to women with isolated hyperglycemia (Q4 vs. Q1: −0.38, 95% CI: −0.91, 0.15) were weaker compared with early childhood exposures, and CIs included null values (Table 4). The strongest association with offspring BMI *z*-score was for maternal second trimester DHA plasma levels, but only among offspring of women with GDM (Q4 vs. Q1: −0.98, 95% CI: −1.89, −0.07). We found potential inverse associations with offspring waist circumference for both cord plasma EPA and DHA in the total cohort and offspring to women with isolated hyperglycemia, but all CIs included unity (Supplement Table 3). Associations with offspring leptin were not consistent in direction and magnitude across the exposures, and CIs were too wide to draw meaningful inference (Supplement Table 4). Cord plasma DHA was associated with lower offspring SS:TR × 100 ratio (Q4 vs. Q1: −3.92%, 95% CI: −7.43%, −0.41%) in the total population (Table 5).

**Table 4:** Multivariable association of prenatal n-3 LCPUFA status and intake with offspring mid-childhood BMI *z*-score, overall and stratified by glucose tolerance status

	**Total**	**Total**	**GDM**	**GIGT**	**IH**	**Normal**
**Exposure**	**Age- and sex-adjusted** ***β*** **(95% CI)**	MV-adjusted^a^ ***β*** **(95% CI)**	MV-adjusted^a^ ***β*** **(95% CI)**	MV-adjusted^a^ ***β*** **(95% CI)**	MV-adjusted^a^ ***β*** **(95% CI)**	MV-adjusted^a^ ***β*** **(95% CI)**
*Continuous exposure*						
Second T plasma EPA (per *z*-score)	−0.04 (−0.12, 0.04)	−0.01 (−0.09, 0.07)	−0.25 (−0.65, 0.15)	0.01 (−1.03, 1.06)	−0.18 (−0.43, 0.07)	0.03 (−0.06, 0.11)
Second T plasma DHA (per *z*-score)	−0.03 (−0.11, 0.04)	−0.01 (−0.09, 0.06)	−0.32 (−0.73, 0.10)	0.12 (−0.43, 0.66)	−0.01 (−0.25, 0.24)	0.00 (−0.08, 0.08)
Cord plasma EPA (per *z*-score)	−0.09 (−0.18, 0.01)	−0.03 (−0.12, 0.05)	−0.06 (−0.61, 0.50)	0.34 (−0.39, 1.06)	−0.07 (−0.30, 0.16)	−0.04 (−0.14, 0.06)
Cord plasma DHA (per *z*-score)	−0.07 (−0.16, 0.02)	−0.05 (−0.13, 0.04)	−0.14 (−0.65, 0.37)	0.25 (−0.36, 0.86)	−0.06 (−0.27, 0.15)	−0.07 (−0.17, 0.03)
DHA + EPA intake (100 mg/day)	−0.04 (−0.08, 0.00)**	−0.02 (−0.06, 0.01)	−0.01 (−0.17, 0.14)	−0.26 (−0.66, 0.13)	−0.08 (−0.25, 0.09)	−0.02 (−0.06, 0.02)
Fish intake (port/week)	0.01 (−0.03, 0.05)	0.02 (−0.02, 0.06)	0.03 (−0.12, 0.19)	−0.18 (−0.64, 0.28)	0.02 (−0.15, 0.19)	0.02 (−0.03, 0.06)
*Categorical exposure*						
Second T plasma EPA						
Q2 vs. Q1	−0.04 (−0.22, 0.15)	0.02 (−0.15, 0.20)	−0.35 (−1.58, 0.89)	0.08 (−0.99, 1.14)	0.00 (−0.68, 0.67)	0.03 (−0.15, 0.22)
Q3 vs. Q1	−0.27 (−0.46, −0.08)**	−0.20 (−0.37, −0.02)**	−1.01 (−2.07, 0.05)	−0.65 (−1.56, 0.27)	0.05 (−0.60, 0.70)	−0.18 (−0.37, 0.00)**
Q4 vs. Q1	−0.15 (−0.33, 0.03)	−0.05 (−0.23, 0.13)	−0.92 (−1.97, 0.13)	0.92 (−0.61, 2.45)	−0.20 (−0.81, 0.40)	0.00 (−0.19, 0.19)
Second T plasma DHA						
Q2 vs. Q1	−0.09 (−0.28, 0.09)	−0.04 (−0.22, 0.13)	−0.24 (−1.27, 0.80)	−0.27 (−2.12, 1.59)	−0.06 (−0.67, 0.56)	−0.03 (−0.22, 0.17)
Q3 vs. Q1	−0.15 (−0.33, 0.03)	−0.10 (−0.27, 0.07)	−1.03 (−2.07, 0.01)	−0.59 (−1.47, 0.29)	0.00 (−0.57, 0.57)	−0.07 (−0.26, 0.12)
Q4 vs. Q1	−0.18 (−0.36, 0.01)	−0.09 (−0.27, 0.08)	−0.98 (−1.89, −0.07)**	0.51 (−0.63, 1.64)	−0.07 (−0.72, 0.57)	−0.06 (−0.25, 0.13)
Cord plasma EPA						
Q2 vs. Q1	−0.10 (−0.29, 0.10)	−0.08 (−0.26, 0.10)	0.72 (−0.75, 2.19)	0.80 (−0.67, 2.27)	−0.25 (−0.92, 0.42)	−0.11 (−0.31, 0.08)
Q3 vs. Q1	−0.04 (−0.24, 0.15)	−0.04 (−0.22, 0.14)	0.04 (−0.89, 0.97)	0.81 (−0.23, 1.84)	−0.15 (−0.75, 0.46)	−0.07 (−0.27, 0.14)
Q4 vs. Q1	−0.23 (−0.42, −0.04)**	−0.13 (−0.32, 0.05)	0.10 (−1.01, 1.21)	−0.08 (−1.52, 1.35)	−0.41 (−0.98, 0.16)	−0.15 (−0.35, 0.05)
Cord plasma DHA						
Q2 vs. Q1	0.00 (−0.20, 0.20)	−0.03 (−0.22, 0.16)	−0.01 (−0.92, 0.90)	0.87 (−0.24, 1.97)	−0.35 (−1.06, 0.35)	−0.03 (−0.23, 0.18)
Q3 vs. Q1	−0.09 (−0.28, 0.10)	−0.09 (−0.27, 0.09)	−0.88 (−2.07, 0.30)	0.74 (−0.38, 1.86)	−0.04 (−0.68, 0.60)	−0.10 (−0.29, 0.10)
Q4 vs. Q1	−0.16 (−0.35, 0.02)	−0.14 (−0.32, 0.04)	0.07 (−1.04, 1.19)	0.33 (−0.80, 1.46)	−0.38 (−0.91, 0.15)	−0.16 (−0.37, 0.04)
DHA + EPA intake						
Q2 vs. Q1	−0.01 (−0.19, 0.17)	−0.01 (−0.18, 0.16)	−0.89 (−2.03, 0.25)	−0.34 (−1.60, 0.92)	−0.01 (−0.65, 0.62)	0.06 (−0.13, 0.24)
Q3 vs. Q1	−0.12 (−0.29, 0.06)	−0.10 (−0.27, 0.07)	−1.17 (−2.23, −0.11)**	0.07 (−0.98, 1.12)	−0.42 (−0.97, 0.13)	−0.03 (−0.22, 0.15)
Q4 vs. Q1	−0.12 (−0.30, 0.06)	−0.07 (−0.24, 0.10)	−0.52 (−1.56, 0.53)	−0.89 (−2.00, 0.22)	−0.17 (−0.77, 0.42)	−0.02 (−0.20, 0.16)
Fish intake						
>0 to <3 vs. 0 port/wk	−0.08 (−0.27, 0.12)	−0.09 (−0.27, 0.10)	−0.87 (−1.93, 0.19)	0.37 (−0.60, 1.34)	0.08 (−0.56, 0.72)	−0.10 (−0.29, 0.10)
≥3 vs. 0 port/wk	0.03 (−0.23, 0.28)	0.02 (−0.22, 0.26)	−0.64 (−1.85, 0.57)	−0.37 (−2.89, 2.15)	0.50 (−0.44, 1.44)	−0.02 (−0.27, 0.24)

*EPA* eicosapentaenoic acid, *DHA* docosahexaenoic acid, *GDM* gestational diabetes mellitus, *GIGT* gestational impaired glucose intolerance, *IH* isolated hyperglycemia, *port* portion, *Q* quartile, *T* trimester

**P < 0.05

^a^Adjusted for child age and sex + maternal education, age, parity, pre-pregnancy BMI, smoking during pregnancy, and household income

**Table 5:** Multivariable association of prenatal n-3 LCPUFA status and intake with offspring mid-childhood SS:TR ratio (SS:TR × 100), overall and stratified by glucose tolerance status

	**Total**	**Total**	**GDM**	**GIGT**	**IH**	**Normal**
**Exposure**	**Age- and sex-adjusted** ***β*** **(95% CI)**	MV-adjusted^a^ ***β*** **(95% CI)**	MV-adjusted^a^ ***β*** **(95% CI)**	MV-adjusted^a^ ***β*** **(95% CI)**	MV-adjusted^a^ ***β*** **(95% CI)**	MV-adjusted^a^ ***β*** **(95% CI)**
*Continuous exposure*						
Second T plasma EPA (per *z*-score)	0.56 (−1.11, 2.22)	1.06 (−0.59, 2.71)	0.60 (−6.58, 7.79)	17.29 (−5.23, 39.82)	3.17 (−1.30, 7.65)	0.45 (−1.33, 2.22)
Second T plasma DHA (per *z*-score)	0.17 (−1.26, 1.61)	0.47 (−0.96, 1.89)	1.66 (−6.34, 9.65)	5.42 (−7.27, 18.11)	1.48 (−2.78, 5.74)	−0.09 (−1.64, 1.47)
Cord plasma EPA (per *z*-score)	−1.51 (−3.22, 0.20)	−1.11 (−2.83, 0.61)	2.55 (−8.97, 14.07)	−10.8 (−38.8, 17.26)	−0.13 (−5.20, 4.94)	−1.67 (−3.53, 0.18)
Cord plasma DHA (per *z*-score)	−1.34 (−3.02, 0.34)	−1.23 (−2.88, 0.41)	0.40 (−10.5, 11.33)	−7.11 (−30.3, 16.11)	−0.21 (−4.81, 4.40)	−1.76 (−3.59, 0.06)
DHA + EPA intake (100 mg/day)	0.77 (0.01, 1.53)**	0.93 (0.18, 1.68)**	1.54 (−1.20, 4.28)	0.00 (−7.88, 7.88)	1.74 (−1.58, 5.06)	0.84 (0.02, 1.67)**
Fish intake (port/week)	0.86 (0.03, 1.69)**	0.95 (0.14, 1.77)**	1.71 (−1.05, 4.47)	0.02 (−9.11, 9.16)	−0.05 (−3.24, 3.15)	0.93 (0.02, 1.85)**
*Categorical exposure*						
Second T plasma EPA						
Q2 vs. Q1	−0.30 (−3.64, 3.04)	0.49 (−2.78, 3.77)	−6.84 (−31.2, 17.49)	5.81 (−16.8, 28.46)	−3.41 (−17.9, 11.06)	0.75 (−2.77, 4.27)
Q3 vs. Q1	−2.15 (−5.50, 1.21)	−0.74 (−4.10, 2.63)	−13.9 (−34.5, 6.81)	−3.42 (−21.8, 14.92)	1.36 (−11.6, 14.28)	−0.71 (−4.32, 2.90)
Q4 vs. Q1	−1.48 (−5.06, 2.10)	0.15 (−3.39, 3.70)	−10.8 (−31.3, 9.57)	33.79 (0.66, 66.93)**	1.78 (−10.2, 13.72)	−0.56 (−4.37, 3.24)
Second T plasma DHA						
Q2 vs. Q1	−0.59 (−3.96, 2.77)	0.17 (−3.20, 3.54)	−1.59 (−23.7, 20.55)	9.23 (−30.5, 48.94)	−1.78 (−14.0, 10.46)	0.24 (−3.35, 3.84)
Q3 vs. Q1	−1.13 (−4.62, 2.35)	−0.06 (−3.59, 3.48)	−3.28 (−24.4, 17.84)	−3.14 (−20.8, 14.49)	−1.10 (−12.9, 10.68)	0.14 (−3.70, 3.98)
Q4 vs. Q1	−1.52 (−4.94, 1.91)	−0.18 (−3.55, 3.20)	−9.74 (−28.0, 8.52)	20.73 (−3.03, 44.49)	0.39 (−12.6, 13.34)	-0.70 (−4.37, 2.96)
Cord plasma EPA						
Q2 vs. Q1	−1.41 (−5.01, 2.19)	−1.27 (−4.77, 2.24)	−3.86 (−33.1, 25.34)	−13.5 (−44.1, 17.16)	−1.52 (−14.9, 11.88)	−0.99 (−4.69, 2.71)
Q3 vs. Q1	−4.13 (−7.76, −0.50)**	−4.20 (−7.75, −0.64)**	−4.14 (−21.3, 13.02)	−14.5 (−36.8, 7.88)	−5.36 (−17.3, 6.54)	−3.68 (−7.60, 0.25)
Q4 vs. Q1	−3.58 (−7.12, −0.04)**	−2.74 (−6.24, 0.76)	13.96 (−7.2, 35.15)	−16.0 (−47.1, 15.03)	−1.69 (−13.1, 9.74)	−3.79 (−7.55, −0.03)**
Cord plasma DHA						
Q2 vs. Q1	−0.96 (−4.64, 2.73)	−1.39 (−4.94, 2.16)	4.50 (−12.7, 21.69)	−12.0 (−35.1, 11.15)	−0.75 (−14.9, 13.40)	−1.83 (−5.69, 2.03)
Q3 vs. Q1	−3.01 (−6.69, 0.67)	−3.22 (−6.80, 0.36)	−11.7 (−36.8, 13.45)	−14.5 (−39.3, 10.32)	−4.23 (−17.2, 8.77)	−2.49 (−6.39, 1.41)
Q4 vs. Q1	−4.01 (−7.62, −0.40)**	−3.92 (−7.43, −0.41)**	6.03 (−15.8, 27.88)	−17.0 (−42.2, 8.18)	−4.43 (−15.2, 6.39)	−4.27 (−8.11, −0.42)**
DHA + EPA intake						
Q2 vs. Q1	−0.28 (−3.75, 3.19)	−0.23 (−3.66, 3.20)	5.11 (−16.8, 27.00)	6.15 (−22.6, 34.86)	2.13 (−10.4, 14.66)	−0.02 (−3.74, 3.70)
Q3 v. Q1	0.55 (−2.91, 4.01)	0.94 (−2.42, 4.30)	−0.50 (−20.1, 19.14)	4.92 (−16.9, 26.77)	6.44 (−4.10, 16.98)	0.59 (−3.10, 4.28)
Q4 vs. Q1	1.37 (−2.08, 4.82)	2.16 (−1.21, 5.54)	5.18 (−14.9, 25.30)	−0.17 (−23.7, 23.33)	4.86 (−6.78, 16.51)	1.98 (−1.70, 5.65)
Fish intake						
>0 to <3 vs. 0 port/wk	−1.11 (−4.91, 2.68)	−0.90 (−4.63, 2.83)	−1.86 (−20.9, 17.18)	−6.29 (−26.5, 13.95)	3.94 (−8.63, 16.51)	−1.54 (−5.61, 2.54)
≥3 vs. 0 port/wk	2.68 (−2.21, 7.56)	2.70 (−2.11, 7.50)	5.77 (−16.5, 28.02)	−0.55 (−26.3, 25.21)	1.65 (−16.1, 19.41)	2.07 (−3.14, 7.28)

*EPA* eicosapentaenoic acid, *DHA* docosahexaenoic acid, *GDM* gestational diabetes mellitus, *GIGT* gestational impaired glucose intolerance, *IH* isolated hyperglycemia, *port* portion, *Q* quartile, *T* trimester.

**P < 0.05

^a^Adjusted for child age and sex + maternal education, age, parity, pre-pregnancy BMI, smoking during pregnancy, and household income

Effect estimates for mid-childhood adiponectin were of greater magnitude compared to early childhood for both DHA + EPA (Q4 vs. Q1: −2.06, 95% CI: −4.18, 0.06 μg/mL) and fish (≥3 vs. 0 portions/week: −2.92, 95% CI: −5.92, 0.08 μg/mL) intake, but CIs were wide (Supplement Table 5). There were trends toward an association for cord plasma DHA (Q4 vs. Q1: −0.12, 95% CI: −0.26, 0.02) and DHA + EPA intake (Q4 vs. Q1: −0.10, 95% CI: −0.24, 0.04) with the offspring metabolic risk score (Supplement table 6). We did not find associations for the other outcomes (data not shown).

## Discussion

In this study, we found an association of cord blood DHA levels with lower offspring BMI and waist circumference in early childhood, which was strongest among offspring to women with isolated hyperglycemia, the mildest form of abnormal glycemia in mid-pregnancy. The directionality remained for the mid-childhood visit, but the associations with BMI and waist circumference were weaker in magnitude by 25–40% and CIs included the null. A greater attenuation (50–95%) across childhood visits was present for offspring leptin levels. Maternal intake of DHA and EPA as well as fish were related to lower offspring adiponectin in early childhood, but the association was attenuated in mid-childhood. We did not see strong evidence for associations of any of the prenatal fatty acids with offspring skinfold and DXA measures, BP, or the metabolic syndrome score.

N-3 LCPUFA intake has previously been found to be associated with higher birth weight, likely mediated by longer gestation^51,52^. While n-3 LCPUFAs were proposed by S.F.O. as the common cause of higher fetal growth and reduced cardiovascular disease over 20 years ago through potentially shared anti-thrombotic mechanisms^53^, the role of these fatty acids in postnatal growth and metabolic health remains poorly understood. Formula supplementation trials in preterm infants have shown that supplementing with DHA alone reduces infant growth (as weight, length, and/or weight:length ratio), while formula with DHA and arachidonic acid normalizes growth patterns and velocity to that of breastfed term infants^54,55^. The few trials that supplemented pregnant women with either EPA and DHA, or DHA alone have found no effect on offspring BMI in infancy;^15,17,56^ one study found a decrease in offspring ponderal index at birth^7^. These studies provided supplements only in the latter half of the pregnancy and only one study^15^ extended postnatal follow-up beyond the first 2 years of life, making it difficult to draw conclusions about the exposure window or long-term effects. Similar lack of associations has been found for observational data, most of which used biomarker measures of either maternal^8,9,12,14^ or cord blood^13,57,58^ or both^31^, and majority had a longer follow-up of 6–7 years. Compared to the present study, most of these studies had smaller sample sizes (*n* < 400), focused on late pregnancy exposure, and did not evaluate other offspring metabolic markers. We previously reported in Project Viva that higher maternal intake and blood, and cord blood concentrations of LCPUFA were associated with lower fetal growth and birth weight^59^, and lower offspring skinfold thickness and risk for obesity in early childhood^31^. In the present study, we found that this association remained present in a larger sample from our cohort, but was attenuated by mid-childhood (median age: 7.7 years). Associations with early childhood adiposity were strongest for cord plasma. In our present study sample maternal and cord plasma EPA (Pearson’s *r* = 0.26) and DHA (Pearson’s *r* = 0.11) levels were weakly-to-moderately correlated. Our findings may therefore imply that cord plasma fatty acids are a better proxy for fetal exposure or that exposure late in pregnancy may be more relevant. It is noteworthy that despite consistent inverse associations with early and mid-childhood BMI, we did not find corresponding reductions in total offspring adiposity measures by skinfold or DXA. However, in the total cohort, inverse relations between cord plasma DHA and offspring SS:TR × 100 ratio were present in early and mid-childhood. This suggest that the effect may lie in changes to central adiposity, a result that was also supported by the inverse association with waist circumference.

The combined evidence from this analysis therefore suggests that prenatal fatty acid may have short-term effects on anthropometry that attenuate as the offspring grow older, perhaps because the child’s own environmental and lifestyle factors have greater influence over time. On the other hand, evidence from the literature suggests that associations of prenatal exposures with adiposity may persist and even strengthen with time. In one study, cord serum n-6:n-3 LCPUFA ratio was associated with lower BMI *z*-scores at age 2, no effect at age 6, and higher BMI *z*-score at age 10^58^. Similarly, prenatal exposure to maternal hyperglycemia^60,61^ has been shown to be associated with higher BMI at birth and in later childhood, but not in early childhood. It is also plausible that prenatal effects may resurface later in life as the metabolic burden increases upon an aging physiology; long-term studies are needed to examine these associations.

We found no associations with offspring glucose, insulin, HOMA-IR, or any of the cholesterol and triglyceride measures, which largely supports the current literature^62^. Courville et al.^7^ found a reduction in cord blood insulin after supplementing DHA in the third trimester. In an observational study, Zhao et al.^63^ found that n-3 LCPUFA in cord plasma was associated with lower proinsulin in cord plasma. It seems likely that any effects of n-3 LCPUFA on glycemia do not persist into childhood. Consistent with our findings, neither prenatal fish oil supplementation nor intake of n-3 LCPUFA was found to influence offspring BP in a randomized trial and cohort study of Danish mother–offspring dyads with 19–20 years’ follow-up^16,64^.

A previous study in Project Viva examined n-3 LCPUFA status in less than half of the currently available cord blood samples (*n* = 424 vs. 153), and did not find an association of prenatal n-3 LCPUFA status with offspring leptin at age 3^31^. With additional fatty acid measurements and an expanded sample size we found tendencies toward lower offspring leptin levels with higher cord plasma EPA and DHA. These results were in agreement with the anthropometry associations as leptin tends to track with adiposity^65^. However, the association with leptin was no longer present in mid-childhood. Notably, maternal DHA + EPA and fish intakes were inversely related to early childhood adiponectin. While CIs for associations of maternal intake of fish and DHA + EPA with BMI *z*-scores and leptin were wide and included the null value, the directionality of the effect estimates were largely similar to those of cord blood DHA with these outcomes. Since in adolescents and adults, cross-sectional lower adiposity has been found to accompany higher adiponectin^66^, the findings seem counter-intuitive. However, adiponectin decreases from birth until at least age 3 before stabilizing or beginning to increase^67,68^. Furthermore, longitudinal animal studies have suggested that adiponectin initially increases alongside body weight and overall fat mass, but levels out and starts to decrease as visceral fat accumulates^69^. It is therefore possible that in this young population adiponectin is still on a downward trajectory and tracking with body weight, providing a possible explanation for the inverse association with higher maternal DHA + EPA and fish intake.

Associations of cord n-3 LCPUFA with offspring BMI *z*-score were stronger in early childhood among offspring of women with isolated hyperglycemia than those of women with normal glucose tolerance, GIGT, or GDM, and at mid-childhood for offspring to women with GDM vs. GIGT/isolated hyperglycemia/normal glycemic status. This is some of the first evidence to suggest a role for dietary factors as mitigators of adverse metabolic consequences in offspring to women with compromised glucose tolerance. In women with both milder and more severe hyperglycemia, n-3 LCPUFA may be acting as insulin-sensitizing agents^27^, reducing maternal hyperglycemia and subsequent fetal hyperinsulinemia driving adiposity accumulation in the offspring^70^. The general lack of associations in offspring of women with GDM and GIGT, which are more progressive states of maternal glucose intolerance, may be due monitoring or treatment of these women as well as dietary or medical interventions altering the level of n-3 LCPUFA or hyperglycemia. However, treatment guidelines during the enrollment period generally did not include GIGT, although this does not preclude that decisions on monitoring or treatment were taken by individual healthcare providers. It should also be noted that both groups had small sample sizes. More well-powered dietary studies of these women and their offspring would further clarify the function of nutritional agents in mitigating adverse metabolic outcomes in the offspring.

There were some limitations to this study. Sample size was small for the models evaluating cord blood measures with some of the outcome biomarkers. However, the strongest associations were still found for cord blood, suggesting a lower exposure measurement error or more proximate exposure to the developing fetus. Nutrient intake reported in the FFQ is subject to measurement error, although the error is more likely to be non-differential given the prospective nature of the study. As such non-differential measurement error would attenuate the effect estimates toward the null. Maternal fatty acids were assessed only in second trimester and we can therefore not rule out effects earlier in pregnancy. While most fetal growth takes place in second and third trimesters and may be more important for anthropometric outcomes, much of the organ architecture is laid down earlier in pregnancy and nutritional influences during this time period may affect physiology. We did not have DXA measurements for early childhood and were not able to directly compare DXA body composition measures between the two time points. The collected data included detailed prospective information on many covariates, including maternal education, pre-preegnancy BMI, smoking, and glucose tolerance status. However, we cannot exclude the possibility of residual confounding. This study included numerous exposure and outcome associations, raising the likelihood of chance findings due to multiple comparisons. Instead of imposing a conservative correction for multiple testing we looked for consistency and strength in findings, as well as biological plausibility. There were some differences in maternal sociodemographic and lifestyle covariates, but not exposures, between mothers with and without outcome data, pointing to limited selection bias. Project Viva participants had health insurance, and many were lege-educated^32^. Our results may therefore not be generalizable to un/underinsured and less-educated populations.

There were several strengths to this study. The prospective, longitudinal design allowed for the comprehensive study of prenatal fatty acid status in relation to cardiometabolic outcomes at two time points in childhood. With outcome data in both early and mid-childhood we could examine whether prenatal effects persisted across a relatively short time period. We included biomarker measures of the exposure as well as of intake to examine whether associations with biomarkers translated to findings in maternal intake. While intake may be more relevant for making recommendations, biomarkers are closer to the physiological pathways of interest. We had detailed outcome data that were measured objectively rather than self-reported and allowed us to examine a broader cardiometabolic profile that included not only classical risk factors such as adiposity, HOMA-IR, and BP but also adipokines.

In this study, we found that higher umbilical cord plasma DHA was associated with lower offspring BMI *z*-scores, waist circumference, and leptin level in early, but not in mid-childhood. These associations tended to be stronger among offspring born to mothers with isolated hyperglycemia. Maternal dietary DHA + EPA and fish intake was inversely related to offspring adiponectin level. This study supports early, limited, and potentially transient effects of prenatal n-3 LCPUFA status on offspring metabolic health, but longitudinal studies of offspring are needed to clarify effects that may emerge over time. Further dietary studies of mother–offspring dyads exposed to prenatal hyperglycemia are warranted.

## Disclaimer

The views expressed in this article do not necessarily represent the views of the US Government, the Department of Health and Human Services or the National Institutes of Health.

## Electronic supplementary material

Supplementary materials
