# Supplementary material for: Prenatal n-3 long-chain fatty acid status and offspring metabolic health in early and mid-childhood: results from Project Viva
Source: Nutr Diabetes. 2018 May 25;8:29. doi: 10.1038/s41387-018-0040-2 (PMC5968023; doi:10.1038/s41387-018-0040-2)
Supplement: Supplementary file 1 — Supplementary materials [file 41387_2018_40_MOESM1_ESM.docx]

Supplementary table 1. Multivariable association of prenatal n-3 LCPUFA status and intake with offspring early childhood leptin levels (ng/mL), overall and stratified by glucose tolerance status

|  | **Total** | **Total** | **GDM** | **GIGT** | **IH** | **Normal** |
| --- | --- | --- | --- | --- | --- | --- |
| **Exposure** | **Age- and sex-adjusted**  **β (95% CI)** | **MV-adjusted***  **β (95% CI)** | **MV-adjusted***  **β (95% CI)** | **MV-adjusted***  **β (95% CI)** | **MV-adjusted***  **β (95% CI)** | **MV-adjusted***  **β (95% CI)** |
| **Continuous exposure** |  |  |  |  |  |  |
| 2nd T plasma EPA (per z-score) | -0.02 (-0.15, 0.12) | 0.00 (-0.13, 0.14) | -0.33 (-1.73, 1.08) | 1.01 (-0.51, 2.53) | -0.18 (-0.66, 0.30) | 0.06 (-0.07, 0.20) |
| 2nd T plasma DHA (per z-score) | -0.02 (-0.16, 0.12) | 0.00 (-0.14, 0.14) | -0.83 (-2.20, 0.55) | 0.41 (-0.43, 1.24) | 0.02 (-0.43, 0.46) | 0.04 (-0.11, 0.19) |
| Cord plasma EPA (per z-score) | -0.11 (-0.30, 0.08) | -0.07 (-0.26, 0.12) | -0.24 (-1.33, 0.85) | -0.87 (-2.24, 0.49) | -0.38 (-0.87, 0.10) | -0.03 (-0.25, 0.19) |
| Cord plasma DHA (per z-score) | -0.15 (-0.34, 0.05) | -0.10 (-0.30, 0.09) | -0.29 (-1.39, 0.81) | -0.60 (-1.54, 0.35) | -0.34 (-0.81, 0.12) | -0.05 (-0.29, 0.18) |
| DHA+EPA intake (100 mg/day) | -0.05 (-0.15, 0.05) | -0.04 (-0.14, 0.05) | 0.23 (-0.46, 0.92) | 0.54 (-0.15, 1.23) | -0.24 (-0.65, 0.17) | -0.06 (-0.15, 0.03) |
| Fish intake (port/week) | -0.04 (-0.17, 0.09) | -0.04 (-0.17, 0.09) | 0.30 (-0.44, 1.05) | 0.42 (-0.37, 1.21) | -0.26 (-0.63, 0.11) | -0.07 (-0.18, 0.04) |
| **Categorical exposure** |  |  |  |  |  |  |
| 2nd T plasma EPA (Q2 v. Q1) | -0.22 (-0.61, 0.16) | -0.16 (-0.53, 0.22) | 1.67 (-3.68, 7.03) | -0.45 (-2.79, 1.89) | 0.09 (-1.49, 1.66) | -0.19 (-0.59, 0.21) |
| (Q3 v. Q1) | -0.35 (-0.74, 0.04) | -0.28 (-0.68, 0.11) | -0.76 (-4.39, 2.87) | -0.34 (-1.81, 1.13) | -0.05 (-1.57, 1.46) | -0.22 (-0.65, 0.21) |
| (Q4 v. Q1) | -0.28 (-0.67, 0.10) | -0.19 (-0.58, 0.21) | -2.41 (-6.39, 1.57) | 0.98 (-1.35, 3.30) | -0.49 (-1.79, 0.81) | -0.06 (-0.49, 0.37) |
| 2nd T plasma DHA (Q2 v. Q1) | -0.21 (-0.60, 0.17) | -0.15 (-0.53, 0.23) | 3.13 (-0.15, 6.42) | -0.13 (-2.97, 2.71) | -0.44 (-1.90, 1.03) | -0.25 (-0.65, 0.16) |
| (Q3 v. Q1) | -0.19 (-0.59, 0.21) | -0.13 (-0.52, 0.26) | -0.65 (-3.95, 2.66) | -0.29 (-1.77, 1.20) | -0.09 (-1.42, 1.25) | -0.10 (-0.53, 0.33) |
| (Q4 v. Q1) | -0.26 (-0.64, 0.11) | -0.15 (-0.53, 0.22) | -0.86 (-4.16, 2.45) | 0.42 (-1.34, 2.18) | 0.11 (-1.27, 1.49) | -0.15 (-0.56, 0.26) |
| Cord plasma EPA (Q2 v. Q1) | -0.23 (-0.65, 0.20) | -0.20 (-0.61, 0.22) | -0.18 (-4.90, 4.54) | -0.82 (-2.92, 1.28) | -0.39 (-1.87, 1.10) | -0.12 (-0.55, 0.32) |
| (Q3 v. Q1) | -0.14 (-0.58, 0.30) | -0.14 (-0.58, 0.29) | -1.24 (-4.58, 2.10) | -1.07 (-3.08, 0.94) | -0.34 (-1.90, 1.22) | -0.05 (-0.52, 0.42) |
| (Q4 v. Q1) | -0.44 (-0.87,-0.01)^**^ | -0.36 (-0.78, 0.06) | -1.83 (-5.12, 1.47) | -0.47 (-3.03, 2.08) | -1.19 (-2.57, 0.19) | -0.21 (-0.67, 0.25) |
| Cord plasma DHA (Q2 v. Q1) | -0.44 (-0.87,-0.02)^**^ | -0.44 (-0.86,-0.03)^**^ | -0.87 (-3.85, 2.12) | -1.10 (-3.34, 1.13) | -0.48 (-1.93, 0.97) | -0.41 (-0.86, 0.04) |
| (Q3 v. Q1) | -0.27 (-0.71, 0.17) | -0.30 (-0.73, 0.13) | -2.60 (-6.77, 1.57) | -0.99 (-3.40, 1.41) | 0.73 (-1.24, 2.69) | -0.26 (-0.71, 0.19) |
| (Q4 v. Q1) | -0.42 (-0.84,-0.01)^**^ | -0.36 (-0.77, 0.05) | -2.67 (-6.85, 1.51) | -0.69 (-2.63, 1.25) | -1.27 (-2.53,-0.02) | -0.17 (-0.62, 0.28) |
| DHA+EPA intake (Q2 v. Q1) | 0.02 (-0.38, 0.42) | -0.01 (-0.40, 0.38) | 0.87 (-2.63, 4.37) | 0.14 (-1.46, 1.74) | -1.24 (-2.80, 0.32) | 0.03 (-0.39, 0.45) |
| (Q3 v. Q1) | -0.14 (-0.55, 0.26) | -0.13 (-0.53, 0.26) | -1.06 (-4.78, 2.66) | 0.52 (-0.96, 2.01) | -1.57 (-2.96,-0.19)^**^ | 0.00 (-0.43, 0.43) |
| (Q4 v. Q1) | -0.21 (-0.61, 0.19) | -0.19 (-0.58, 0.21) | -0.27 (-4.14, 3.59) | 1.88 (-0.13, 3.89) | -1.22 (-2.58, 0.14) | -0.18 (-0.60, 0.24) |
| Fish intake (>0 to <3 v. 0 port/wk) | -0.25 (-0.69, 0.18) | -0.25 (-0.68, 0.17) | -0.17 (-3.41, 3.06) | -0.42 (-1.71, 0.86) | -1.35 (-3.04, 0.34) | -0.18 (-0.65, 0.29) |
| (>=3 v. 0 port/wk) | -0.45 (-1.05, 0.16) | -0.47 (-1.06, 0.12) | -0.02 (-3.98, 3.93) | 0.00 (-0.92, 0.92) | -1.70 (-3.90, 0.50) | -0.43 (-1.04, 0.19) |

EPA: eicosapentaenoic acid; DHA: docosahexaenoic acid; GDM: gestational diabetes mellitus; GIGT: gestational impaired glucose intolerance; IH: isolated hyperglycemia; Port: portion; Q: quartile; T: trimester.

^*^Adjusted for child age and sex + maternal education, age, parity, pre-pregnancy BMI, smoking during pregnancy and household income.

^**^P<0.05

Supplementary table 2. Multivariable association of prenatal n-3 LCPUFA status and intake with offspring early childhood adiponectin (µg/mL), overall and stratified by glucose tolerance status

|  | **Total** | **Total** | **GDM** | **GIGT** | **IH** | **Normal** |
| --- | --- | --- | --- | --- | --- | --- |
| **Exposure** | **Age- and sex-adjusted**  **β (95% CI)** | **MV-adjusted***  **β (95% CI)** | **MV-adjusted***  **β (95% CI)** | **MV-adjusted***  **β (95% CI)** | **MV-adjusted***  **β (95% CI)** | **MV-adjusted***  **β (95% CI)** |
| **Continuous exposure** |  |  |  |  |  |  |
| 2nd T plasma EPA (per z-score) | -0.30 (-0.75, 0.15) | -0.35 (-0.81, 0.10) | -0.66 (-3.86, 2.55) | 0.30 (-6.02, 6.62) | 0.37 (-1.21, 1.95) | -0.43 (-0.92, 0.06) |
| 2nd T plasma DHA (per z-score) | -0.34 (-0.83, 0.15) | -0.36 (-0.86, 0.13) | -0.41 (-2.91, 2.09) | 1.13 (-1.95, 4.20) | 0.18 (-1.21, 1.56) | -0.43 (-0.98, 0.13) |
| Cord plasma EPA (per z-score) | -0.19 (-0.81, 0.44) | -0.24 (-0.88, 0.40) | -0.80 (-4.85, 3.25) | 1.25 (-8.55, 11.05) | 0.29 (-1.42, 2.00) | -0.35 (-1.09, 0.39) |
| Cord plasma DHA (per z-score) | -0.22 (-0.88, 0.44) | -0.23 (-0.91, 0.44) | -0.85 (-4.66, 2.95) | 0.03 (-7.26, 7.32) | 0.43 (-1.14, 2.00) | -0.33 (-1.15, 0.48) |
| DHA+EPA intake (100 mg/day) | -0.42 (-0.70,-0.14)^**^ | -0.47 (-0.75,-0.18)^**^ | 0.10 (-1.42, 1.63) | -0.54 (-3.17, 2.08) | -0.51 (-1.71, 0.69) | -0.48 (-0.76,-0.20)^**^ |
| Fish intake (port/week) | -0.35 (-0.67,-0.04)^**^ | -0.42 (-0.73,-0.10)^**^ | 0.40 (-0.75, 1.54) | -0.57 (-3.66, 2.53) | -0.90 (-2.04, 0.23) | -0.40 (-0.74,-0.06)^**^ |
| **Categorical exposure** |  |  |  |  |  |  |
| 2nd T plasma EPA (Q2 v. Q1) | 0.05 (-1.19, 1.29) | -0.03 (-1.27, 1.22) | 1.42 (-8.84, 11.67) | 1.45 (-7.17, 10.07) | 0.05 (-4.47, 4.56) | -0.14 (-1.50, 1.22) |
| (Q3 v. Q1) | -0.15 (-1.43, 1.13) | -0.35 (-1.63, 0.94) | 0.43 (-6.33, 7.19) | 4.62 (-0.33, 9.57) | 0.35 (-4.11, 4.82) | -0.61 (-2.02, 0.81) |
| (Q4 v. Q1) | -0.49 (-1.77, 0.78) | -0.79 (-2.08, 0.49) | -3.29 (-11.5, 4.91) | -3.42 (-12.1, 5.22) | 1.77 (-2.10, 5.65) | -1.03 (-2.44, 0.37) |
| 2nd T plasma DHA (Q2 v. Q1) | -0.05 (-1.30, 1.21) | -0.15 (-1.40, 1.10) | 1.30 (-4.62, 7.23) | 3.83 (-4.95, 12.61) | -0.68 (-5.35, 3.98) | -0.23 (-1.59, 1.13) |
| (Q3 v. Q1) | 0.05 (-1.25, 1.35) | -0.01 (-1.31, 1.30) | -2.39 (-8.82, 4.05) | 4.16 (-1.27, 9.60) | -0.22 (-4.29, 3.85) | 0.00 (-1.49, 1.50) |
| (Q4 v. Q1) | -0.66 (-1.93, 0.61) | -0.88 (-2.15, 0.40) | -0.89 (-7.68, 5.91) | 1.61 (-4.47, 7.68) | 0.73 (-3.62, 5.07) | -1.13 (-2.50, 0.23) |
| Cord plasma EPA (Q2 v. Q1) | 0.35 (-0.94, 1.63) | 0.26 (-1.02, 1.55) | -3.00 (-10.7, 4.66) | -0.76 (-8.74, 7.23) | 0.38 (-4.12, 4.88) | 0.25 (-1.15, 1.65) |
| (Q3 v. Q1) | 0.51 (-0.86, 1.88) | 0.55 (-0.82, 1.92) | -3.43 (-8.86, 1.99) | 2.78 (-5.23, 10.79) | -0.97 (-5.59, 3.64) | 0.73 (-0.79, 2.26) |
| (Q4 v. Q1) | 0.24 (-1.10, 1.57) | 0.16 (-1.18, 1.50) | -4.21 (-10.2, 1.81) | -1.68 (-15.5, 12.11) | 1.49 (-2.66, 5.63) | 0.20 (-1.34, 1.73) |
| Cord plasma DHA (Q2 v. Q1) | 0.53 (-0.76, 1.82) | 0.47 (-0.82, 1.75) | -2.35 (-7.40, 2.70) | -3.44 (-12.6, 5.72) | -1.43 (-5.89, 3.03) | 0.62 (-0.82, 2.05) |
| (Q3 v. Q1) | -0.08 (-1.51, 1.34) | -0.07 (-1.50, 1.36) | -5.43 (-12.0, 1.10) | 4.80 (-5.75, 15.35) | -0.86 (-6.76, 5.03) | 0.11 (-1.44, 1.67) |
| (Q4 v. Q1) | 0.22 (-1.09, 1.53) | 0.19 (-1.13, 1.50) | -6.15 (-13.2, 0.84) | -1.58 (-10.7, 7.53) | 0.81 (-2.85, 4.46) | 0.29 (-1.24, 1.82) |
| DHA+EPA intake (Q2 v. Q1) | -0.59 (-1.78, 0.60) | -0.64 (-1.83, 0.54) | 0.50 (-5.68, 6.67) | 2.17 (-4.52, 8.86) | 1.01 (-3.91, 5.92) | -0.95 (-2.28, 0.37) |
| (Q3 v. Q1) | -0.67 (-1.93, 0.58) | -0.79 (-2.04, 0.45) | -0.41 (-7.08, 6.26) | -0.98 (-7.20, 5.24) | -0.39 (-4.77, 3.99) | -0.70 (-2.05, 0.64) |
| (Q4 v. Q1) | -1.39 (-2.60,-0.18)^**^ | -1.59 (-2.80,-0.38)^**^ | 0.59 (-6.62, 7.81) | -2.69 (-10.8, 5.45) | -0.94 (-5.20, 3.31) | -1.69 (-3.01,-0.37)^**^ |
| Fish intake (>0 to <3 v. 0 port/wk) | -1.46 (-2.76,-0.17)^**^ | -1.58 (-2.88,-0.28)^**^ | 0.11 (-5.47, 5.69) | -1.11 (-6.15, 3.93) | 0.79 (-4.09, 5.66) | -1.84 (-3.27,-0.40)^**^ |
| (>=3 v. 0 port/wk) | -1.98 (-3.70,-0.25)^**^ | -2.18 (-3.90,-0.47)^**^ | 0.13 (-6.53, 6.80) | -0.15 (-3.27, 2.97) | -2.41 (-9.20, 4.37) | -2.09 (-3.97,-0.21)^**^ |

EPA: eicosapentaenoic acid; DHA: docosahexaenoic acid; GDM: gestational diabetes mellitus; GIGT: gestational impaired glucose intolerance; IH: isolated hyperglycemia; Port: portion; Q: quartile; T: trimester.

^*^Adjusted for child age and sex + maternal education, age, parity, pre-pregnancy BMI, smoking during pregnancy and household income.

^**^P<0.05

Supplementary table 3. Multivariable association of prenatal n-3 LCPUFA status and intake with offspring mid-childhood waist circumference (cm), overall and stratified by glucose tolerance status

|  | **Total** | **Total** | **GDM** | **GIGT** | **IH** | **Normal** |
| --- | --- | --- | --- | --- | --- | --- |
| **Exposure** | **Age- and sex-adjusted**  **β (95% CI)** | **MV-adjusted***  **β (95% CI)** | **MV-adjusted***  **β (95% CI)** | **MV-adjusted***  **β (95% CI)** | **MV-adjusted***  **β (95% CI)** | **MV-adjusted***  **β (95% CI)** |
| **Continuous exposure** |  |  |  |  |  |  |
| 2nd T plasma EPA (per z-score) | -0.01 (-0.64, 0.62) | 0.19 (-0.41, 0.79) | -1.36 (-4.55, 1.82) | 0.98 (-6.02, 7.98) | -1.16 (-3.04, 0.73) | 0.42 (-0.21, 1.04) |
| 2nd T plasma DHA (per z-score) | 0.04 (-0.55, 0.62) | 0.15 (-0.41, 0.71) | -1.79 (-5.12, 1.55) | 1.28 (-2.45, 5.01) | 0.25 (-1.50, 2.01) | 0.18 (-0.40, 0.76) |
| Cord plasma EPA (per z-score) | -0.69 (-1.38, 0.00)^**^ | -0.41 (-1.08, 0.26) | -1.75 (-6.80, 3.29) | 1.49 (-2.43, 5.40) | -0.80 (-2.55, 0.96) | -0.39 (-1.12, 0.34) |
| Cord plasma DHA (per z-score) | -0.37 (-1.05, 0.30) | -0.26 (-0.91, 0.39) | -2.14 (-6.93, 2.65) | 1.15 (-2.19, 4.50) | -0.53 (-2.12, 1.05) | -0.28 (-1.00, 0.45) |
| DHA+EPA intake (100 mg/day) | -0.15 (-0.47, 0.18) | -0.07 (-0.37, 0.24) | 0.19 (-1.16, 1.54) | -0.84 (-3.15, 1.48) | -0.35 (-1.64, 0.95) | -0.07 (-0.41, 0.27) |
| Fish intake (port/week) | 0.18 (-0.18, 0.53) | 0.20 (-0.14, 0.53) | 0.80 (-0.55, 2.15) | 0.24 (-2.46, 2.95) | 0.24 (-1.04, 1.51) | 0.10 (-0.27, 0.47) |
| **Categorical exposure** |  |  |  |  |  |  |
| 2nd T plasma EPA (Q2 v. Q1) | 0.04 (-1.33, 1.40) | 0.41 (-0.92, 1.73) | -0.13 (-11.8, 11.58) | -1.14 (-8.32, 6.04) | 0.56 (-4.43, 5.55) | 0.42 (-0.98, 1.82) |
| (Q3 v. Q1) | -0.98 (-2.41, 0.44) | -0.48 (-1.83, 0.86) | -6.45 (-16.0, 3.05) | -1.50 (-7.36, 4.36) | 0.94 (-3.78, 5.66) | -0.49 (-1.93, 0.94) |
| (Q4 v. Q1) | -0.48 (-1.92, 0.96) | 0.18 (-1.20, 1.56) | -5.81 (-15.3, 3.71) | 4.81 (-5.07, 14.69) | -1.56 (-6.09, 2.97) | 0.53 (-0.94, 1.99) |
| 2nd T plasma DHA (Q2 v. Q1) | -0.36 (-1.76, 1.04) | -0.04 (-1.39, 1.30) | -1.33 (-11.4, 8.71) | -0.44 (-13.3, 12.41) | -0.11 (-4.73, 4.50) | 0.00 (-1.46, 1.46) |
| (Q3 v. Q1) | -0.76 (-2.14, 0.61) | -0.47 (-1.80, 0.85) | -5.56 (-15.0, 3.87) | -1.90 (-7.31, 3.51) | 0.96 (-3.36, 5.28) | -0.52 (-1.97, 0.93) |
| (Q4 v. Q1) | -0.59 (-2.06, 0.89) | -0.02 (-1.45, 1.40) | -6.73 (-15.0, 1.51) | 3.54 (-3.92, 11.00) | 0.81 (-3.95, 5.56) | 0.11 (-1.39, 1.61) |
| Cord plasma EPA (Q2 v. Q1) | -0.78 (-2.29, 0.72) | -0.64 (-2.06, 0.78) | 3.48 (-9.92, 16.88) | 3.26 (-5.86, 12.37) | -1.51 (-6.44, 3.43) | -0.82 (-2.32, 0.68) |
| (Q3 v. Q1) | -0.61 (-2.12, 0.89) | -0.57 (-1.99, 0.85) | -0.02 (-8.30, 8.26) | 2.51 (-3.96, 8.98) | -0.17 (-4.66, 4.32) | -0.91 (-2.47, 0.65) |
| (Q4 v. Q1) | -1.53 (-3.01,-0.06)^**^ | -0.89 (-2.31, 0.52) | 0.65 (-9.36, 10.66) | -1.20 (-10.1, 7.74) | -2.98 (-7.17, 1.22) | -0.94 (-2.46, 0.58) |
| Cord plasma DHA (Q2 v. Q1) | -0.58 (-2.13, 0.96) | -0.83 (-2.28, 0.62) | 0.10 (-8.01, 8.20) | 4.21 (-2.74, 11.15) | -1.80 (-7.01, 3.42) | -1.06 (-2.63, 0.51) |
| (Q3 v. Q1) | -0.97 (-2.45, 0.52) | -0.89 (-2.30, 0.52) | -7.91 (-18.2, 2.42) | 1.96 (-5.13, 9.06) | -0.35 (-5.14, 4.45) | -0.89 (-2.41, 0.63) |
| (Q4 v. Q1) | -0.78 (-2.23, 0.66) | -0.62 (-2.01, 0.76) | 0.36 (-9.96, 10.69) | 0.69 (-6.33, 7.70) | -2.19 (-6.18, 1.80) | -0.72 (-2.24, 0.81) |
| DHA+EPA intake (Q2 v. Q1) | -0.37 (-1.81, 1.06) | -0.45 (-1.82, 0.92) | -9.04 (-18.5, 0.40) | -2.76 (-9.91, 4.39) | 0.07 (-4.59, 4.72) | 0.11 (-1.36, 1.58) |
| (Q3 v. Q1) | -1.42 (-2.81,-0.02)^**^ | -1.36 (-2.69,-0.03)^**^ | -9.93 (-19.2,-0.69)^**^ | 2.63 (-3.81, 9.07) | -2.92 (-6.90, 1.06) | -1.02 (-2.48, 0.44) |
| (Q4 v. Q1) | -0.87 (-2.30, 0.56) | -0.60 (-1.97, 0.77) | -4.94 (-14.1, 4.16) | -3.06 (-9.47, 3.36) | -0.16 (-4.56, 4.24) | -0.43 (-1.92, 1.06) |
| Fish intake (>0 to <3 v. 0 port/wk) | -0.69 (-2.26, 0.88) | -0.81 (-2.29, 0.66) | -7.70 (-16.7, 1.26) | 3.82 (-1.74, 9.39) | 1.06 (-3.70, 5.81) | -1.00 (-2.61, 0.60) |
| (>=3 v. 0 port/wk) | 0.05 (-1.98, 2.08) | -0.12 (-2.06, 1.81) | -4.17 (-14.6, 6.31) | -0.81 (-9.34, 7.73) | 2.95 (-4.15, 10.06) | -0.62 (-2.68, 1.44) |

EPA: eicosapentaenoic acid; DHA: docosahexaenoic acid; GDM: gestational diabetes mellitus; GIGT: gestational impaired glucose intolerance; IH: isolated hyperglycemia; Port: portion; Q: quartile; T: trimester.

^*^Adjusted for child age and sex + maternal education, age, parity, pre-pregnancy BMI, smoking during pregnancy and household income.

^**^P<0.05

Supplementary table 4. Multivariable association of prenatal n-3 LCPUFA status and intake with offspring mid-childhood leptin (ng/mL), overall and stratified by glucose tolerance status

|  | **Total** | **Total** | **GDM** | **GIGT** | **IH** | **Normal** |
| --- | --- | --- | --- | --- | --- | --- |
| **Exposure** | **Age- and sex-adjusted**  **β (95% CI)** | **MV-adjusted***  **β (95% CI)** | **MV-adjusted***  **β (95% CI)** | **MV-adjusted***  **β (95% CI)** | **MV-adjusted***  **β (95% CI)** | **MV-adjusted***  **β (95% CI)** |
| **Continuous exposure** |  |  |  |  |  |  |
| 2nd T plasma EPA (per z-score) | 0.48 (-0.35, 1.31) | 0.60 (-0.24, 1.44) | -0.37 (-7.91, 7.17) | 0.61 (-11.2, 12.41) | -0.76 (-3.44, 1.92) | 0.75 (-0.16, 1.66) |
| 2nd T plasma DHA (per z-score) | 0.30 (-0.32, 0.92) | 0.35 (-0.28, 0.97) | 0.13 (-4.48, 4.74) | 0.27 (-5.85, 6.39) | 0.07 (-2.37, 2.51) | 0.33 (-0.33, 0.99) |
| Cord plasma EPA (per z-score) | 0.21 (-0.69, 1.10) | 0.25 (-0.68, 1.17) | 0.29 (-5.61, 6.18) | 0.88 (-5.44, 7.19) | 0.79 (-1.95, 3.53) | -0.13 (-1.26, 0.99) |
| Cord plasma DHA (per z-score) | 0.22 (-0.55, 0.98) | 0.19 (-0.60, 0.98) | -0.78 (-3.49, 1.93) | 0.93 (-3.80, 5.66) | 0.77 (-1.78, 3.31) | -0.08 (-1.05, 0.88) |
| DHA+EPA intake (100 mg/day) | 0.07 (-0.30, 0.45) | 0.09 (-0.28, 0.46) | 1.01 (-0.11, 2.13) | -1.84 (-5.42, 1.74) | -1.18 (-2.92, 0.55) | 0.03 (-0.37, 0.44) |
| Fish intake (port/week) | 0.28 (-0.14, 0.69) | 0.32 (-0.09, 0.73) | 1.11 (0.19, 2.04) | -1.43 (-5.84, 2.99) | -0.58 (-2.58, 1.41) | 0.21 (-0.27, 0.68) |
| **Categorical exposure** |  |  |  |  |  |  |
| 2nd T plasma EPA (Q2 v. Q1) | 0.27 (-1.30, 1.84) | 0.58 (-0.99, 2.15) | 9.10 (-3.55, 21.76) | -0.54 (-10.6, 9.53) | 2.61 (-6.69, 11.91) | 0.02 (-1.61, 1.65) |
| (Q3 v. Q1) | 0.03 (-1.54, 1.59) | 0.47 (-1.11, 2.04) | 2.54 (-5.41, 10.49) | -2.25 (-11.1, 6.63) | 1.35 (-5.48, 8.17) | 0.28 (-1.47, 2.03) |
| (Q4 v. Q1) | 0.57 (-1.07, 2.22) | 1.12 (-0.54, 2.79) | -1.70 (-10.6, 7.18) | 6.14 (-13.6, 25.92) | 1.66 (-4.91, 8.23) | 0.83 (-1.00, 2.66) |
| 2nd T plasma DHA (Q2 v. Q1) | 0.29 (-1.30, 1.88) | 0.51 (-1.07, 2.08) | 4.21 (-4.67, 13.08) | -8.58 (-23.8, 6.67) | -1.31 (-8.85, 6.23) | 0.42 (-1.30, 2.13) |
| (Q3 v. Q1) | 0.67 (-0.98, 2.31) | 0.91 (-0.75, 2.56) | 0.97 (-8.56, 10.50) | 0.60 (-5.62, 6.83) | 3.01 (-3.59, 9.61) | 0.37 (-1.46, 2.20) |
| (Q4 v. Q1) | 0.12 (-1.48, 1.72) | 0.52 (-1.09, 2.13) | 0.92 (-6.40, 8.24) | 2.37 (-8.91, 13.65) | 1.84 (-4.97, 8.65) | 0.27 (-1.47, 2.02) |
| Cord plasma EPA (Q2 v. Q1) | -0.15 (-1.83, 1.52) | -0.20 (-1.85, 1.46) | 1.73 (-10.9, 14.35) | -1.32 (-13.3, 10.66) | -3.66 (-11.4, 4.10) | 0.18 (-1.58, 1.95) |
| (Q3 v. Q1) | -0.15 (-1.83, 1.53) | -0.31 (-1.98, 1.35) | 3.82 (-3.85, 11.49) | 0.53 (-11.2, 12.27) | -1.57 (-8.03, 4.89) | -0.43 (-2.27, 1.41) |
| (Q4 v. Q1) | 0.10 (-1.77, 1.97) | 0.17 (-1.73, 2.07) | -1.20 (-10.6, 8.23) | -4.74 (-16.8, 7.34) | 0.04 (-5.93, 6.02) | 0.06 (-2.17, 2.28) |
| Cord plasma DHA (Q2 v. Q1) | -0.72 (-2.43, 0.99) | -0.79 (-2.49, 0.91) | 3.84 (-3.39, 11.07) | 2.67 (-7.08, 12.43) | -4.93 (-12.3, 2.44) | -0.51 (-2.39, 1.36) |
| (Q3 v. Q1) | 0.59 (-1.18, 2.37) | 0.40 (-1.36, 2.15) | -5.04 (-15.5, 5.37) | -2.28 (-13.0, 8.43) | 0.03 (-7.85, 7.92) | 0.39 (-1.50, 2.27) |
| (Q4 v. Q1) | 0.01 (-1.66, 1.68) | -0.18 (-1.87, 1.51) | -0.69 (-8.98, 7.60) | -1.16 (-9.20, 6.89) | -0.40 (-6.01, 5.21) | -0.29 (-2.26, 1.67) |
| DHA+EPA intake (Q2 v. Q1) | -1.56 (-3.27, 0.15) | -1.48 (-3.19, 0.22) | -2.34 (-14.1, 9.46) | -0.27 (-8.52, 7.97) | 0.18 (-7.39, 7.74) | -1.16 (-3.00, 0.67) |
| (Q3 v. Q1) | -0.44 (-2.16, 1.28) | -0.42 (-2.12, 1.29) | -1.21 (-10.9, 8.43) | -3.09 (-11.4, 5.18) | -1.55 (-7.73, 4.64) | -0.19 (-2.09, 1.71) |
| (Q4 v. Q1) | -0.66 (-2.40, 1.07) | -0.54 (-2.27, 1.18) | -0.13 (-8.89, 8.64) | -5.40 (-16.7, 5.91) | -4.32 (-10.4, 1.79) | -0.28 (-2.17, 1.61) |
| Fish intake (>0 to <3 v. 0 port/wk) | 0.14 (-1.82, 2.11) | 0.03 (-1.92, 1.98) | -2.21 (-11.3, 6.85) | -11.0 (-28.7, 6.72) | 2.86 (-4.68, 10.41) | -0.03 (-2.17, 2.11) |
| (>=3 v. 0 port/wk) | 0.34 (-2.12, 2.79) | 0.31 (-2.14, 2.75) | 0.04 (-8.84, 8.93) | -3.14 (-24.6, 18.34) | 1.53 (-9.29, 12.35) | -0.04 (-2.71, 2.64) |

EPA: eicosapentaenoic acid; DHA: docosahexaenoic acid; GDM: gestational diabetes mellitus; GIGT: gestational impaired glucose intolerance; IH: isolated hyperglycemia; Port: portion; Q: quartile; T: trimester.

^*^Adjusted for child age and sex + maternal education, age, parity, pre-pregnancy BMI, smoking during pregnancy and household income.

^**^P<0.05

Supplementary table 5. Multivariable association of prenatal n-3 LCPUFA status and intake with offspring mid-childhood adiponectin (µU/mL), overall and stratified by glucose tolerance status

|  | **Total** | **Total** | **GDM** | **GIGT** | **IH** | **Normal** |
| --- | --- | --- | --- | --- | --- | --- |
| **Exposure** | **Age- and sex-adjusted**  **β (95% CI)** | **MV-adjusted***  **β (95% CI)** | **MV-adjusted***  **β (95% CI)** | **MV-adjusted***  **β (95% CI)** | **MV-adjusted***  **β (95% CI)** | **MV-adjusted***  **β (95% CI)** |
| **Continuous exposure** |  |  |  |  |  |  |
| 2nd T plasma EPA (per z-score) | -0.41 (-1.56, 0.73) | -0.52 (-1.68, 0.64) | -5.51 (-11.9, 0.84) | 2.46 (-9.97, 14.89) | -0.92 (-4.69, 2.85) | -0.56 (-1.85, 0.74) |
| 2nd T plasma DHA (per z-score) | -0.55 (-1.51, 0.41) | -0.60 (-1.56, 0.36) | -2.58 (-6.25, 1.09) | 2.28 (-3.27, 7.84) | -1.75 (-5.34, 1.84) | -0.49 (-1.50, 0.52) |
| Cord plasma EPA (per z-score) | 0.45 (-0.60, 1.49) | 0.35 (-0.72, 1.42) | 1.36 (-8.07, 10.80) | 1.21 (-9.64, 12.05) | -0.96 (-4.22, 2.30) | 0.34 (-0.91, 1.59) |
| Cord plasma DHA (per z-score) | 0.44 (-0.50, 1.38) | 0.41 (-0.56, 1.37) | -0.12 (-4.35, 4.10) | 0.25 (-8.37, 8.88) | -0.53 (-3.52, 2.46) | 0.44 (-0.70, 1.58) |
| DHA+EPA intake (100 mg/day) | -0.35 (-0.78, 0.08) | -0.40 (-0.83, 0.04) | -0.87 (-1.96, 0.23) | 6.06 (0.33, 11.79) | -1.01 (-3.89, 1.86) | -0.33 (-0.80, 0.14) |
| Fish intake (port/week) | -0.33 (-0.80, 0.15) | -0.36 (-0.85, 0.12) | -0.77 (-1.73, 0.19) | 4.82 (-2.50, 12.14) | -1.75 (-5.04, 1.54) | -0.22 (-0.76, 0.32) |
| **Categorical exposure** |  |  |  |  |  |  |
| 2nd T plasma EPA (Q2 v. Q1) | -0.27 (-2.27, 1.74) | -0.43 (-2.47, 1.61) | -2.63 (-12.7, 7.48) | -6.54 (-23.8, 10.67) | 6.68 (-7.12, 20.47) | -0.56 (-2.66, 1.54) |
| (Q3 v. Q1) | -1.25 (-3.37, 0.88) | -1.43 (-3.58, 0.73) | -1.28 (-9.08, 6.52) | -1.64 (-17.4, 14.09) | -2.77 (-13.3, 7.77) | -1.43 (-3.73, 0.87) |
| (Q4 v. Q1) | -0.40 (-2.61, 1.81) | -0.70 (-2.93, 1.53) | -9.26 (-18.7, 0.18) | -11.2 (-35.1, 12.59) | 0.65 (-9.34, 10.64) | -0.83 (-3.15, 1.48) |
| 2nd T plasma DHA (Q2 v. Q1) | -0.30 (-2.54, 1.94) | -0.35 (-2.59, 1.89) | -3.26 (-10.9, 4.36) | 2.58 (-20.4, 25.58) | 3.31 (-9.10, 15.72) | -0.53 (-2.85, 1.79) |
| (Q3 v. Q1) | -0.85 (-3.15, 1.45) | -0.97 (-3.28, 1.35) | -5.82 (-14.4, 2.80) | -2.67 (-14.5, 9.11) | -1.17 (-12.4, 10.07) | -0.71 (-3.09, 1.67) |
| (Q4 v. Q1) | -0.80 (-2.99, 1.39) | -1.02 (-3.23, 1.20) | -5.51 (-12.4, 1.39) | -2.61 (-17.6, 12.43) | 0.59 (-10.5, 11.71) | -1.06 (-3.34, 1.23) |
| Cord plasma EPA (Q2 v. Q1) | -0.58 (-2.73, 1.56) | -0.61 (-2.76, 1.53) | 2.17 (-10.4, 14.74) | -13.6 (-35.2, 8.07) | 1.93 (-9.55, 13.42) | -0.98 (-3.22, 1.26) |
| (Q3 v. Q1) | 0.00 (-2.17, 2.17) | 0.00 (-2.18, 2.19) | -2.79 (-9.89, 4.31) | -2.69 (-19.4, 14.02) | -1.84 (-11.6, 7.91) | 0.39 (-2.04, 2.82) |
| (Q4 v. Q1) | 1.23 (-1.11, 3.56) | 1.30 (-1.06, 3.66) | -4.96 (-14.8, 4.85) | -0.72 (-19.5, 18.09) | 0.47 (-9.01, 9.94) | 1.58 (-1.05, 4.20) |
| Cord plasma DHA (Q2 v. Q1) | 0.17 (-2.05, 2.40) | 0.14 (-2.09, 2.38) | -0.32 (-7.50, 6.85) | -15.0 (-29.4,-0.59)^**^ | 0.68 (-10.5, 11.82) | 0.12 (-2.33, 2.57) |
| (Q3 v. Q1) | -0.77 (-3.00, 1.46) | -0.85 (-3.11, 1.41) | -2.37 (-12.9, 8.17) | 4.17 (-11.8, 20.14) | -3.56 (-15.6, 8.45) | -0.45 (-2.85, 1.96) |
| (Q4 v. Q1) | 0.99 (-1.09, 3.08) | 1.12 (-0.99, 3.23) | -4.28 (-12.9, 4.34) | -7.87 (-19.9, 4.19) | 0.83 (-7.96, 9.62) | 1.45 (-0.91, 3.81) |
| DHA+EPA intake (Q2 v. Q1) | -1.37 (-3.56, 0.82) | -1.47 (-3.66, 0.73) | 0.83 (-9.09, 10.74) | 1.56 (-11.0, 14.11) | -1.89 (-16.4, 12.63) | -1.72 (-4.02, 0.58) |
| (Q3 v. Q1) | -0.74 (-2.94, 1.46) | -0.84 (-3.04, 1.36) | 2.00 (-5.81, 9.80) | 5.33 (-7.34, 18.01) | 1.99 (-9.53, 13.51) | -1.47 (-3.82, 0.88) |
| (Q4 v. Q1) | -1.88 (-3.98, 0.22) | -2.06 (-4.18, 0.06) | -5.48 (-12.7, 1.72) | 14.51 (-3.59,32.62) | -3.97 (-14.6, 6.60) | -2.03 (-4.28, 0.23) |
| Fish intake (>0 to <3 v. 0 port/wk) | -2.36 (-4.84, 0.13) | -2.38 (-4.86, 0.11) | 0.36 (-8.03, 8.74) | 0.60 (-25.7, 26.87) | -2.00 (-15.4, 11.39) | -2.36 (-4.96, 0.25) |
| (>=3 v. 0 port/wk) | -2.84 (-5.83, 0.14) | -2.92 (-5.92, 0.08) | -3.86 (-12.1, 4.43) | 1.13 (-17.9, 20.11) | -7.02 (-25.0, 10.92) | -2.29 (-5.47, 0.89) |

EPA: eicosapentaenoic acid; DHA: docosahexaenoic acid; GDM: gestational diabetes mellitus; GIGT: gestational impaired glucose intolerance; IH: isolated hyperglycemia; Port: portion; Q: quartile; T: trimester.

^*^Adjusted for child age and sex + maternal education, age, parity, pre-pregnancy BMI, smoking during pregnancy and household income.

^**^P<0.05

Supplementary table 6. Multivariable association of prenatal n-3 LCPUFA status and intake with offspring mid-childhood metabolic risk score, overall and stratified by glucose tolerance status

|  | **Total** | **Total** | **GDM** | **GIGT** | **IH** | **Normal** |
| --- | --- | --- | --- | --- | --- | --- |
| **Exposure** | **Age- and sex-adjusted**  **β (95% CI)** | **MV-adjusted***  **β (95% CI)** | **MV-adjusted***  **β (95% CI)** | **MV-adjusted***  **β (95% CI)** | **MV-adjusted***  **β (95% CI)** | **MV-adjusted***  **β (95% CI)** |
| **Continuous exposure** |  |  |  |  |  |  |
| 2nd T plasma EPA (per z-score) | 0.01 (-0.06, 0.08) | 0.02 (-0.05, 0.09) | 0.17 (-0.54, 0.88) | 0.58 (-0.65, 1.81) | -0.12 (-0.26, 0.02) | 0.03 (-0.04, 0.11) |
| 2nd T plasma DHA (per z-score) | -0.02 (-0.08, 0.04) | -0.02 (-0.08, 0.04) | -0.08 (-0.50, 0.34) | 0.17 (-0.49, 0.83) | -0.07 (-0.21, 0.07) | -0.01 (-0.08, 0.05) |
| Cord plasma EPA (per z-score) | -0.01 (-0.08, 0.06) | -0.01 (-0.08, 0.06) | -0.19 (-1.55, 1.16) | -0.36 (-1.07, 0.34) | -0.03 (-0.21, 0.15) | -0.02 (-0.11, 0.06) |
| Cord plasma DHA (per z-score) | -0.02 (-0.09, 0.04) | -0.03 (-0.09, 0.04) | -0.20 (-0.80, 0.40) | -0.33 (-0.73, 0.07) | -0.04 (-0.21, 0.13) | -0.05 (-0.13, 0.03) |
| DHA+EPA intake (100 mg/day) | -0.01 (-0.04, 0.02) | -0.01 (-0.04, 0.02) | 0.03 (-0.11, 0.17) | -0.06 (-0.54, 0.41) | -0.03 (-0.16, 0.11) | -0.02 (-0.05, 0.01) |
| Fish intake (port/week) | 0.01 (-0.02, 0.04) | 0.01 (-0.02, 0.04) | 0.06 (-0.06, 0.18) | 0.07 (-0.51, 0.65) | -0.04 (-0.19, 0.11) | 0.00 (-0.04, 0.03) |
| **Categorical exposure** |  |  |  |  |  |  |
| 2nd T plasma EPA (Q2 v. Q1) | 0.03 (-0.11, 0.17) | 0.06 (-0.08, 0.19) | 0.35 (-1.15, 1.84) | -0.12 (-1.80, 1.57) | -0.17 (-0.75, 0.41) | 0.07 (-0.07, 0.22) |
| (Q3 v. Q1) | -0.03 (-0.18, 0.11) | 0.00 (-0.14, 0.14) | 0.05 (-1.03, 1.13) | 0.05 (-1.36, 1.46) | 0.01 (-0.45, 0.46) | -0.01 (-0.16, 0.14) |
| (Q4 v. Q1) | -0.01 (-0.16, 0.13) | 0.03 (-0.11, 0.17) | 0.06 (-1.13, 1.26) | 0.95 (-0.84, 2.74) | -0.18 (-0.59, 0.24) | 0.03 (-0.12, 0.18) |
| 2nd T plasma DHA (Q2 v. Q1) | -0.01 (-0.15, 0.13) | 0.00 (-0.14, 0.14) | -0.09 (-1.23, 1.05) | 0.63 (-1.14, 2.40) | -0.14 (-0.63, 0.35) | 0.01 (-0.14, 0.15) |
| (Q3 v. Q1) | -0.04 (-0.19, 0.10) | -0.04 (-0.19, 0.11) | -0.32 (-1.47, 0.83) | -0.31 (-1.15, 0.53) | 0.10 (-0.36, 0.55) | -0.06 (-0.22, 0.10) |
| (Q4 v. Q1) | -0.09 (-0.24, 0.05) | -0.05 (-0.20, 0.09) | -0.11 (-1.01, 0.78) | 0.42 (-0.56, 1.39) | -0.17 (-0.63, 0.28) | -0.05 (-0.20, 0.10) |
| Cord plasma EPA (Q2 v. Q1) | -0.15 (-0.29, 0.00) | -0.14 (-0.28, 0.00) | 0.87 (-0.65, 2.39) | 0.48 (-1.17, 2.12) | -0.01 (-0.53, 0.50) | -0.17 (-0.32,-0.02)^**^ |
| (Q3 v. Q1) | -0.04 (-0.18, 0.11) | -0.05 (-0.19, 0.09) | 0.47 (-0.40, 1.34) | 0.04 (-1.45, 1.52) | -0.14 (-0.58, 0.30) | -0.10 (-0.25, 0.06) |
| (Q4 v. Q1) | -0.08 (-0.23, 0.08) | -0.07 (-0.22, 0.09) | -0.08 (-1.17, 1.01) | -0.19 (-1.85, 1.47) | -0.03 (-0.45, 0.39) | -0.10 (-0.28, 0.07) |
| Cord plasma DHA (Q2 v. Q1) | -0.13 (-0.28, 0.02) | -0.14 (-0.29, 0.01) | 0.59 (-0.27, 1.44) | 0.04 (-1.37, 1.44) | -0.17 (-0.66, 0.32) | -0.19 (-0.35,-0.03)^**^ |
| (Q3 v. Q1) | -0.02 (-0.17, 0.13) | -0.03 (-0.18, 0.12) | -0.59 (-1.81, 0.63) | 0.16 (-1.27, 1.59) | 0.18 (-0.35, 0.71) | -0.07 (-0.22, 0.09) |
| (Q4 v. Q1) | -0.11 (-0.26, 0.03) | -0.12 (-0.26, 0.02) | 0.05 (-0.93, 1.03) | -0.20 (-1.32, 0.92) | -0.19 (-0.58, 0.20) | -0.16 (-0.33, 0.00) |
| DHA+EPA intake (Q2 v. Q1) | -0.13 (-0.27, 0.02) | -0.12 (-0.26, 0.02) | -1.01 (-2.30, 0.28) | -0.46 (-1.58, 0.65) | 0.25 (-0.28, 0.78) | -0.09 (-0.24, 0.06) |
| (Q3 v. Q1) | -0.15 (-0.29, 0.00) | -0.14 (-0.28, 0.00) | -0.84 (-1.91, 0.23) | -0.07 (-1.21, 1.07) | -0.24 (-0.65, 0.18) | -0.11 (-0.26, 0.04) |
| (Q4 v. Q1) | -0.11 (-0.25, 0.03) | -0.10 (-0.24, 0.04) | -0.55 (-1.53, 0.42) | -0.07 (-1.55, 1.41) | 0.06 (-0.36, 0.48) | -0.11 (-0.26, 0.04) |
| Fish intake (>0 to <3 v. 0 port/wk) | -0.14 (-0.30, 0.03) | -0.14 (-0.30, 0.02) | -0.88 (-1.87, 0.11) | -0.05 (-2.01, 1.92) | 0.02 (-0.48, 0.53) | -0.14 (-0.32, 0.03) |
| (>=3 v. 0 port/wk) | -0.07 (-0.27, 0.14) | -0.08 (-0.28, 0.12) | -0.30 (-1.29, 0.69) | -0.08 (-1.55, 1.38) | 0.13 (-0.67, 0.93) | -0.14 (-0.36, 0.08) |

EPA: eicosapentaenoic acid; DHA: docosahexaenoic acid; GDM: gestational diabetes mellitus; GIGT: gestational impaired glucose intolerance; IH: isolated hyperglycemia; Port: portion; Q: quartile; T: trimester.

^*^Adjusted for child age and sex + maternal education, age, parity, pre-pregnancy BMI, smoking during pregnancy and household income.

^**^P<0.05
